# Encapsulation of carbon dots in medicinal chemistry: a comprehensive review of recent advances and applications

**DOI:** 10.1039/d5ra09695b

**Published:** 2026-03-17

**Authors:** Sewara J. Mohammed, Kosar M. Ali, Fryad S. Mustafa, Hastyar H. Najmuldeen, Fayez Alghofaili, Mohammed K. Sidiq, Kawan F. Kayani

**Affiliations:** a Department of Chemistry, College of Science, University of Sulaimani Qlyasan Street Sulaymaniyah 46001 Kurdistan Regional Government Iraq sewara.mohammed@univsul.edu.iq; b College of Medicine, University of Sulaimani Qlyasan Street Sulaymaniyah 46001 Kurdistan Regional Government Iraq; c Pharmacy Department, Komar University of Science and Technology Sulaimani 46001 Kurdistan Region Iraq; d Department of Medical Laboratory Analysis, College of Health Sciences, Cihan University Sulaimaniya Sulaymaniyah Kurdistan Iraq; e Department of Biology, College of Science, University of Sulaimani Qlyasan Street Sulaymaniyah 46001 Kurdistan Regional Government Iraq; f Department of Medical Laboratory Sciences, College of Applied Medical Sciences, Majmaah University Majmaah 11952 Saudi Arabia; g Department of Pharmacognosy and Pharmaceutical Chemistry, College of Pharmacy, University of Sulaimani Sulaymaniyah Kurdistan Regional Government 46001 Iraq

## Abstract

Carbon dots (CDs) have emerged as highly promising nanomaterials in medicinal chemistry due to their unique optical properties, excellent biocompatibility, and facile surface functionalization. However, their clinical translation is often hindered by limitations such as colloidal instability, nonspecific biodistribution, and limited therapeutic efficacy. This review emphasizes the pivotal role of encapsulation strategies in overcoming these challenges and enhancing CD functionality for advanced nanomedicine. We provide a comprehensive analysis of various encapsulation techniques, including polymeric, lipid-based, inorganic, and hybrid systems, detailing their mechanisms, advantages, and limitations. Advances in CD synthesis, functionalization, and physicochemical and biophysical characterization are discussed, along with their expanding applications in drug delivery, bioimaging, theranostics, biosensing, and related biomedical fields. Finally, we examine key translational barriers and propose future opportunities in intelligent nanocarrier engineering, clinical development, and regulatory advancement. This review offers a critical and in-depth perspective on encapsulated CDs as innovative, multifunctional platforms poised to advance modern medicinal chemistry.

## Abstract 2

Encapsulation strategies enhance the stability, targeting, and therapeutic performance of carbon dots, enabling advanced applications in drug delivery, bioimaging, biosensing, and theranostics for next-generation nanomedicine.

## Graphical abstract

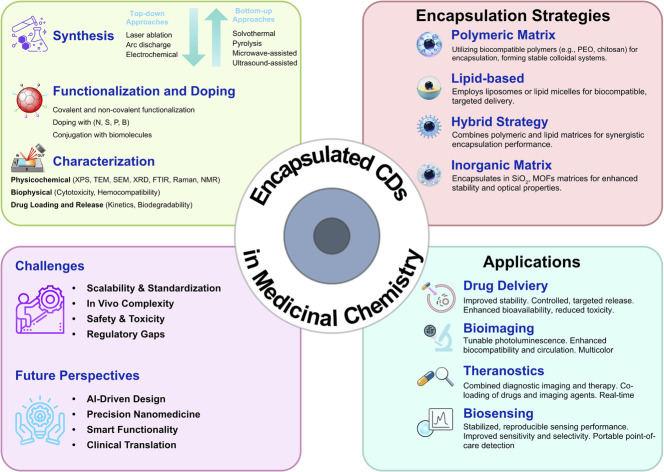

## 1 Introduction

The burgeoning field of nanomedicine holds significant promise for addressing complex healthcare challenges, yet its progress is often constrained by the limitations of conventional nanomaterials.^1^ Carbon dots (CDs), fluorescent carbon-based nanoparticles typically less than 10 nm in size, have emerged as a promising alternative due to their strong and tunable photoluminescence, high water solubility, abundant surface functional groups, and excellent biocompatibility.^2–4^ Unlike semiconductor quantum dots that commonly contain toxic heavy metals, CDs offer a safer and more sustainable profile for biomedical use.^5^ These advantages enable their effective use in advanced bioimaging, targeted drug delivery, sensitive biosensing, and integrated theranostic platforms.^6–9^

Since their serendipitous discovery in 2004 during the purification of single-walled carbon nanotubes,^10^ CDs have evolved from simple fluorescent markers into highly engineered nanomaterials with precisely tailored physicochemical and biological properties.^10^ Advances in synthesis methods, heteroatom doping, and surface functionalization have enabled fine control over particle size, emission behavior, reactivity, and biological interactions, which has greatly expanded their relevance in medicinal chemistry.^8,11,12^

**Fig. 1:**
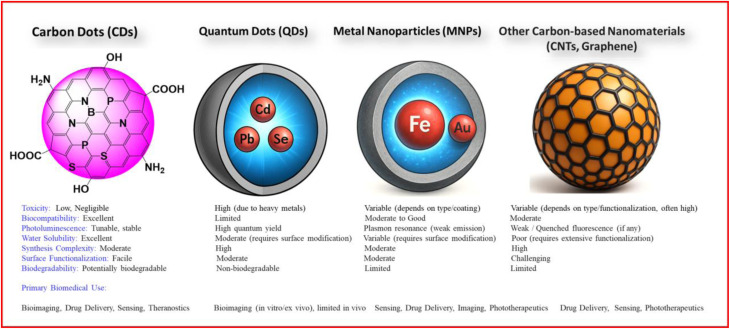
Comparative analysis of CDs with other nanomaterials in biomedical applications.

Within the broader landscape of nanotechnology, CDs offer several advantages over commonly used nanomaterials (Fig. 1). Quantum dots (QDs) provide strong emission but raise concerns because of their heavy-metal content.^13^ Metal nanoparticles (MNPs) often show limited biostability and potential bioaccumulation.^14^ Carbon nanotubes (CNTs) and graphene derivatives face challenges related to hydrophobicity, functionalization complexity, and inconsistent toxicological profiles.^15^ CDs address many of these issues through scalable and environmentally friendly synthesis, excellent aqueous dispersibility, and facile surface modification.^16,17^

Despite these benefits, several barriers continue to hinder the translational progress of CDs. In physiological environments, CDs may experience colloidal instability,^18^ rapid photobleaching,^19^ nonspecific biodistribution,^20^ and limited drug-loading capacity.^21^ These issues can reduce imaging quality, therapeutic performance, and overall *in vivo* reliability. Therefore, strategies that enhance the structural stability, optical robustness, and biological behavior of CDs are essential for enabling practical biomedical use.

**Fig. 2:**
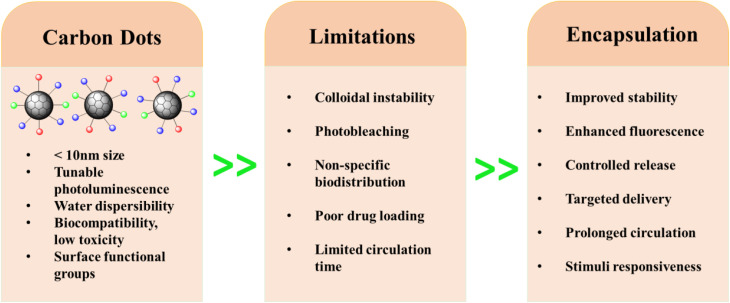
Schematic illustration of CDs, their limitations, and the role of encapsulation in enhancing properties for biomedical applications.

Encapsulation has emerged as a transformative strategy to address these limitations (Fig. 2). Embedding CDs within polymeric matrices, lipid-based vesicles, or inorganic shells can improve colloidal stability, extend systemic circulation time, and reduce optical degradation.^22–24^ Encapsulation can also enhance quantum yield, narrow emission profiles, and increase resistance to quenching.^25^ These improvements are critical for achieving high-resolution bioimaging. In many systems, the encapsulating materials additionally provide stimulus responsiveness, such as pH sensitivity,^26,27^ temperature sensitivity,^28^ light activation, or enzymatic activation, which enables controlled and on-demand therapeutic release.^29,30^

**Fig. 3:**
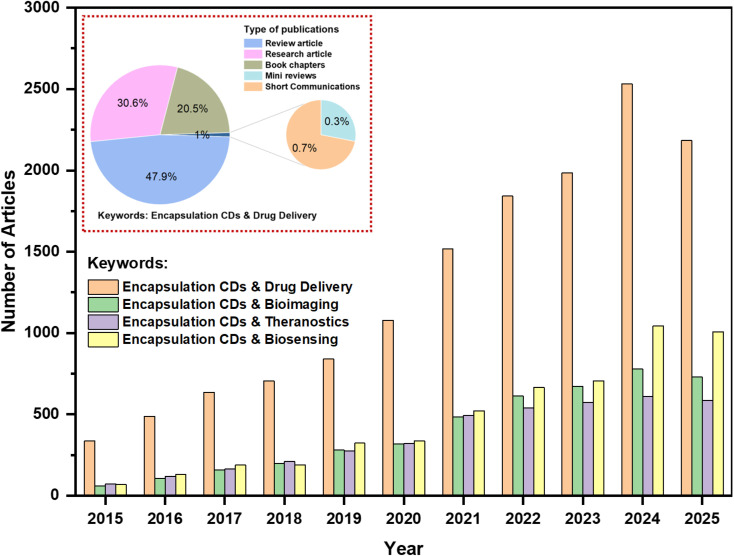
Annual publications on “Encapsulation of Carbon Dots in Drug Delivery, Bioimaging, Theranostics, and Biosensing” from 2015 to 2025. Data were retrieved from ScienceDirect in June 2025 and illustrate the growing research interest in encapsulated CDs for biomedical applications.

The growing scientific interest in this field is reflected in publication trends (Fig. 3). A search of ScienceDirect using the terms “Encapsulation Carbon Dots and Drug Delivery, Bioimaging, Theranostics, and Biosensing” reveals a marked increase in research activity from 2015 to 2025, emphasizing both rapid progress and the need for a comprehensive and integrative assessment. Data were retrieved in June 2025.

This review provides a comprehensive and critical evaluation of recent advances in the encapsulation of CDs for medicinal chemistry. We first examine the synthesis, functionalization, and characterization of CDs as a foundation for understanding encapsulation design. Subsequently, we analyze major encapsulation strategies, polymeric, lipid-based, inorganic, and emerging hybrid systems, discussing their design principles and performance. The review then highlights state-of-the-art applications in drug delivery, bioimaging, theranostics, and biosensing, culminating in a discussion of key translational challenges and future research directions aimed at accelerating the clinical integration of encapsulated CDs.

## 2 Synthesis, functionalization, and characterization of CDs

A clear understanding of the structural classes, synthesis routes, and surface functionalization of CDs is essential for designing effective encapsulated systems. The properties established during CD formation determine their compatibility with different matrices, loading behavior, and performance in biological settings. This section outlines these foundational features and highlights their relevance to rational encapsulation strategies in medicinal chemistry.

### 2.1 Structural types of CDs

**Fig. 4:**
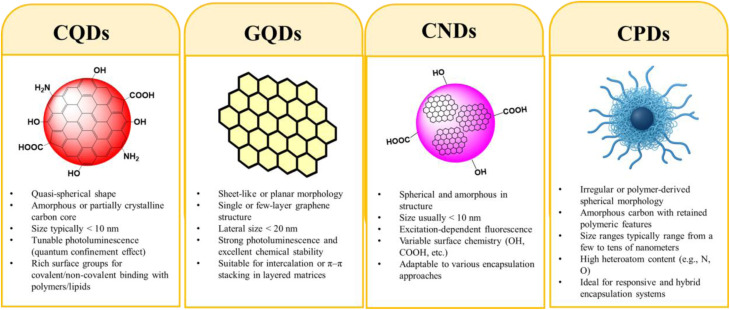
Schematic representation and structural comparison of various CDs, highlighting their distinct morphologies, internal structures, and surface chemistries relevant to encapsulation.

CDs comprise several structural subtypes, each exhibiting distinct morphologies, crystallographic features, and surface chemistries that govern their encapsulation behavior and suitability for biomedical applications, as illustrated in Fig. 4. Carbon Quantum Dots (CQDs) are quasi-spherical nanoparticles (<10 nm) with amorphous or partially crystalline cores and strong, tunable photoluminescence arising from quantum confinement effects.^31^ Their surfaces contain abundant hydroxyl, carboxyl, and amino groups, enabling facile conjugation and compatibility with both polymeric and lipid-based encapsulation systems.^32^

Graphene Quantum Dots (GQDs) consist of one or a few layers of graphene with lateral dimensions typically below 20 nm.^33^ Their high surface area, sp^2^-hybridized lattice, and planar structure allow strong π–π interactions and efficient incorporation into layered matrices or inorganic shells, making them attractive for hybrid encapsulation designs.^34^

Carbon nanodots (CNDs), in contrast, are amorphous particles under 10 nm in size with heterogeneous surface states and excitation-dependent fluorescence, offering versatility across different encapsulation environments.^35^ Carbonized polymer dots (CPDs), produced from polymer precursors, often retain partial polymeric character and exhibit high heteroatom content.^36^ This hybrid nature supports the development of stimuli-responsive encapsulation systems and enhances interactions with soft matrices.

Overall, the structural diversity among CD subtypes directly affects loading capacity, colloidal stability, and release behavior within encapsulating materials. CQDs favor covalent or non-covalent incorporation, while GQDs integrate effectively into layered hosts. The versatile surface chemistries of CNDs and CPDs support innovative hybrid or responsive delivery systems.

### 2.2 Synthesis approaches

**Fig. 5:**
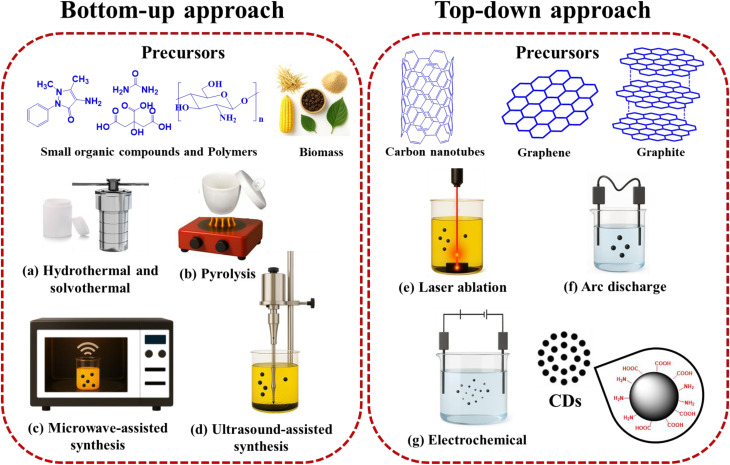
Common synthetic precursors and methods for CDs production, categorized into bottom-up and top-down approaches.

The synthesis of CDs broadly follows bottom-up and top-down strategies, each offering distinct advantages for controlling particle size, crystallinity, surface chemistry, and ultimately their performance within encapsulation systems. Fig. 5 illustrates commonly used precursors and synthetic methods for CD production, classified under these two main strategies.

#### 2.2.1 Bottom-up approaches

Bottom-up approaches involve the construction of CDs from molecular precursors, enabling precise control over nanostructure and surface functionality during synthesis. Among these, hydrothermal and solvothermal syntheses (Fig. 5a) are the most widely employed methods.^5,37^ Both techniques involve the thermal treatment of carbon-rich precursors (*e.g.*, citric acid, saccharides, or biomass) under sealed, high-pressure conditions, which facilitates controlled nucleation and growth. The primary distinction lies in the solvent system: hydrothermal synthesis uses water, offering an environmentally friendly route that typically produces CDs enriched with oxygen-containing functional groups for excellent aqueous solubility. In contrast, solvothermal synthesis uses organic solvents (*e.g.*, ethanol, DMF), enabling finer modulation of physicochemical properties and facilitating doping strategies, making it suitable for generating hydrophobic CDs for integration into non-polar systems. While hydrothermal methods are lauded for their simplicity and scalability, solvothermal approaches offer superior tunability.^38^

Pyrolysis (Fig. 5b) is another common bottom-up method involving the thermal decomposition of organic precursors at high temperatures (>300 °C) in an inert atmosphere. This solid-state process typically yields graphitized CDs with high crystallinity and strong photoluminescence.^39^ While scalable and solvent-free, the resulting CDs often require post-synthesis functionalization to achieve optimal dispersibility for encapsulation.

Microwave-assisted synthesis (Fig. 5c) utilizes microwave radiation to rapidly and uniformly heat carbon precursors, enabling energy-efficient and time-saving CD formation. This method offers fine control over reaction conditions, often yielding monodisperse CDs with narrow size distributions and tunable fluorescence properties, which is attractive for applications like drug delivery and sensing.^8,40^ Similarly, ultrasound-assisted synthesis (Fig. 5d) employs high-frequency ultrasonic waves to induce acoustic cavitation, generating localized high temperatures and pressures that promote CD formation under mild conditions without the need for high temperatures or harsh chemicals.^41,42^ This method is particularly suited for green synthesis from biomass, though it may require longer reaction times and yields lower quantities compared to thermal methods.

From an encapsulation standpoint, bottom-up methods provide CDs with tailored surface chemistries that enhance integration into polymeric, lipidic, or inorganic matrices.

#### 2.2.2 Top-down approaches

Top-down methods generate CDs by breaking down bulk carbon materials, often yielding highly crystalline structures. Laser ablation (Fig. 5e), for instance, employs a high-energy laser beam to irradiate a carbon target (*e.g.*, graphite) in a liquid medium, ejecting carbon clusters that form CDs.^43,44^ This technique offers excellent control over particle size and produces high-purity products, but its scalability is limited by the need for specialized equipment. The arc discharge method (Fig. 5f) uses an electric arc between graphite electrodes to create carbonaceous fragments that reorganize into CDs with high crystallinity.^45^ However, surface functionalities are less tunable and often require post-modification to suit encapsulation systems.

Electrochemical synthesis (Fig. 5g) oxidizes or exfoliates carbon sources under controlled potentials. By adjusting applied voltage and electrolyte composition, CDs with tunable size, charge, and surface groups can be obtained.^46^ This method is particularly advantageous for producing CDs pre-functionalized with charged species, improving compatibility with ionic encapsulation matrices or electrostatically assembled carriers.

Overall, top-down strategies are valuable for producing crystalline CDs with well-defined cores, but often require secondary functionalization to optimize interactions with encapsulating materials.

### 2.3 Surface functionalization and doping

**Fig. 6:**
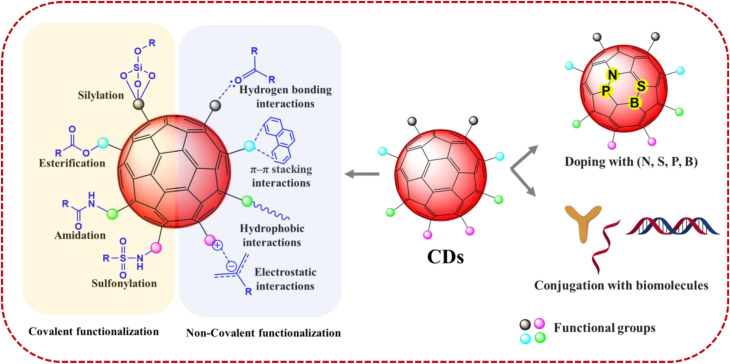
Schematic representation of surface functionalization and doping strategies of CDs.

The surface chemistry of CDs plays a critical role in determining their colloidal stability, biocompatibility, and interactions with biological systems or encapsulating materials.^47,48^ Strategic modification of this surface is therefore essential for tailoring CDs to specific biomedical applications. Fig. 6 illustrates the various surface functionalization and doping strategies employed to tailor the physicochemical properties of CDs for targeted applications.

Surface functionalization is primarily achieved through covalent and non-covalent strategies. Covalent functionalization involves the formation of strong chemical bonds between the CD surface and functional groups or molecules. Common strategies, such as esterification, amidation, sulfonylation, and silanization, leverage the abundant hydroxyl and carboxyl groups present on CD surfaces.^49^ This approach provides robust and stable modifications, which are essential for preventing leaching in encapsulated systems and ensuring long-term performance in biological environments. In contrast, non-covalent strategies, including hydrogen bonding, electrostatic interactions, π–π stacking, or van der Waals forces, offer less disruptive modifications that preserve intrinsic photophysical properties.^50^ Such reversible interactions are advantageous in dynamic self-assembling encapsulation systems, including liposomes and responsive polymer matrices.^51^

Beyond surface attachment, the internal structure of CDs can be modified through heteroatom doping. This process introduces elements such as nitrogen, sulfur, phosphorus, or boron into the carbon framework, enabling precise tuning of optical, electronic, and catalytic properties.^51^ While nitrogen doping enhances fluorescence quantum yield, photostability, and electron-donating capacity,^52^ the introduction of sulfur, phosphorus, or boron can create new defect states and tailor charge transfer behavior.^53,54^ These modifications are particularly beneficial for encapsulated CDs used in deep-tissue imaging, biosensing, triggered drug release, or ROS-mediated therapies, where they enhance critical photophysical performance.^55–60^

To impart biological specificity and advanced functionality, CDs are often conjugated with biomolecules.^61^ Antibody conjugation enables highly targeted delivery to specific cell types or tissues, such as cancer cells expressing a particular antigen.^62^ Peptides can be used to enhance receptor-mediated uptake, promote cell penetration, or provide enzyme-responsive release features.^63^ Nucleotides, including DNA/RNA aptamers and siRNA, play critical roles in gene delivery and biosensing applications.^64^ Encapsulation of these bioconjugated CDs not only protects fragile biomolecules from degradation but also enhances delivery precision and therapeutic efficacy by stabilizing the hybrid construct throughout transport and release.

### 2.4 Characterization techniques

**Fig. 7:**
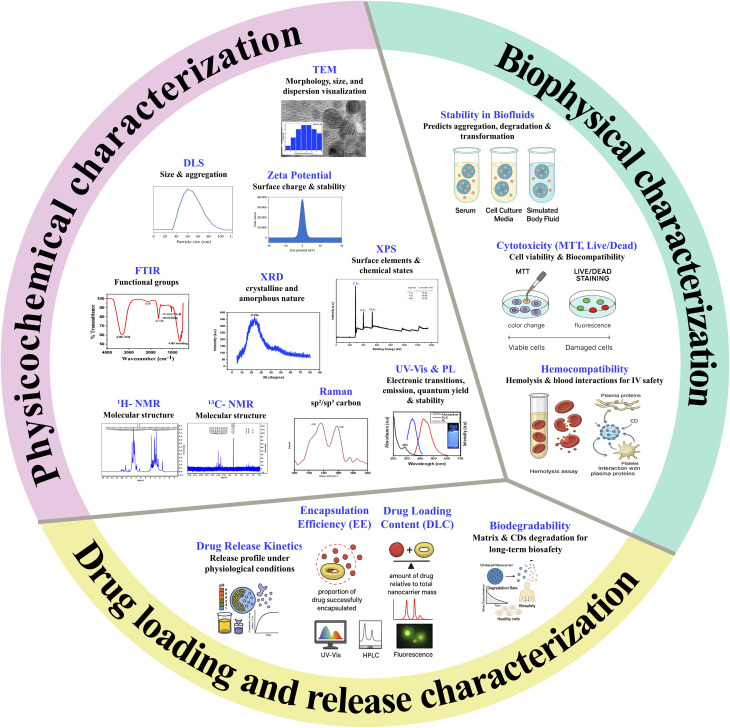
Summary of key characterization techniques for CDs and encapsulated CDs.

Comprehensive characterization of CDs, both in their free and encapsulated forms, is essential for assessing physicochemical properties, stability, and biocompatibility. Fig. 7 provides an overview of the key techniques commonly employed for this purpose, categorized into physicochemical, biophysical, and drug release characterization.

#### 2.4.1 Physicochemical characterization loading

Physicochemical characterization focuses on the intrinsic properties of CDs and their encapsulated counterparts. Transmission electron microscopy (TEM) provides direct visualization of particle morphology, size distribution, and, in encapsulated systems, the core–shell architecture and CD dispersion within matrices.^16,65^ Dynamic light scattering (DLS) evaluates hydrodynamic size and aggregation behavior, while zeta potential analysis provides insights into surface charge and colloidal stability, which are crucial for predicting *in vivo* behavior.^66^ Fourier transform infrared spectroscopy (FTIR) identifies surface functional groups on CDs and their encapsulants, confirming chemical compatibility and successful modification.^67^

X-ray diffraction (XRD) reveals the crystalline or amorphous nature of CDs and encapsulating materials, providing insight into graphitic ordering and structural integrity.^68,69^ X-ray photoelectron spectroscopy (XPS) determines elemental composition and bonding states, enabling verification of heteroatom doping or conjugation.^65^ Nuclear magnetic resonance (NMR) spectroscopy, especially ^1^H-NMR and ^13^C-NMR, offers detailed information on molecular structures and surface chemistry.^65,70^ Raman spectroscopy distinguishes between sp^2^/sp^3^ hybridized carbon, defects, and crystalline structure.^71^

Finally, optical characterization through Ultraviolet-visible (UV-Vis) absorption and photoluminescence (PL) spectroscopy is essential for evaluating band-gap transitions, emission behavior, and photostability.^71,72^ Because encapsulation may influence fluorescence efficiency, these measurements are important for systems designed for imaging or theranostics.

#### 2.4.2 Biophysical characterization

Biophysical techniques assess how CDs and their encapsulated forms behave in biological environments. Evaluating dispersion behavior in physiological fluids (*e.g.*, serum, culture media, simulated body fluids) helps predict aggregation, surface transformations, or premature release from carriers.^73^ Cytotoxicity assays, such as MTT and live/dead staining, assess cell viability and membrane integrity, supporting safety evaluations for therapeutic use.^74,75^

Hemocompatibility tests, including hemolysis assays and interaction studies with plasma proteins or platelets, are critical for evaluating safety in intravenous or systemic delivery applications.^76,77^ Together, these techniques ensure that encapsulated CD systems are not only functional but also safe for biomedical use.

#### 2.4.3 Drug loading and release characterization

For drug delivery applications, it is essential to quantify how efficiently drugs are incorporated into the CD-based nanocarrier and how they are released. Encapsulation efficiency (EE) measures the proportion of drug successfully encapsulated, while drug loading content (DLC) quantifies the amount of drug relative to the total nanocarrier mass. These are typically assessed using UV-Vis spectroscopy, high-performance liquid chromatography (HPLC), or fluorescence analysis.^78^ Drug release kinetics, studied under various physiological conditions (*e.g.*, pH variations, enzyme presence), reveal the release profile and enable the design of controlled or sustained delivery systems.^79–81^ Additionally, evaluating the biodegradability of both the encapsulating matrix and, when relevant, the CDs themselves is critical for long-term biosafety.^82,83^ This involves monitoring the degradation rate *in vitro* or *in vivo* to ensure clearance and minimize toxicity over time.

## 3 Encapsulation strategies for CDs

**Fig. 8:**
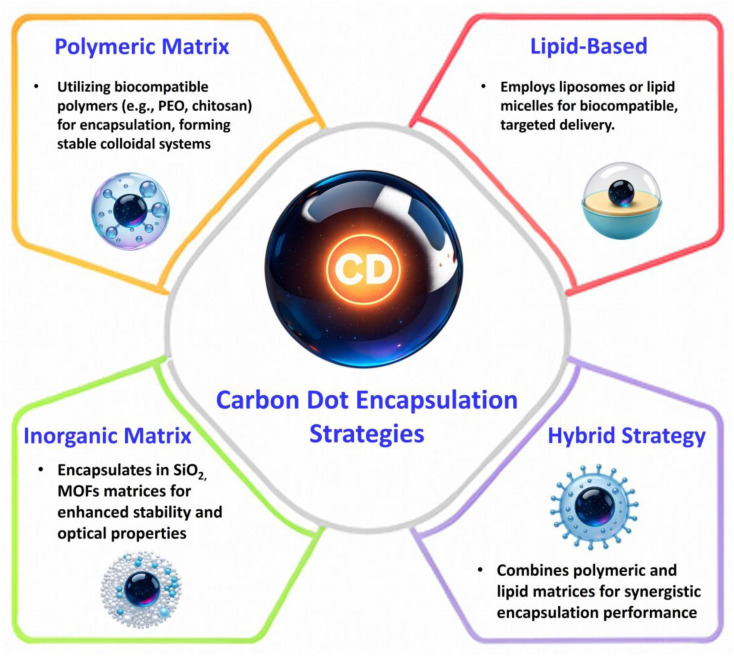
Schematic illustration of major encapsulation strategies for CDs, including polymeric, lipid-based, inorganic, and hybrid systems.

As illustrated in Fig. 8, the major encapsulation strategies employed in CD-based nanomedicine include polymeric, lipid-based, inorganic, and emerging hybrid systems. Each category provides distinct physicochemical advantages; however, their practical performance depends on how effectively these advantages are balanced against limitations related to stability, scalability, and translational feasibility.

To enable cross-platform comparison, this review evaluates encapsulation strategies using standardized criteria: (i) encapsulation efficiency and drug-loading capacity; (ii) physiological and serum stability; (iii) biodegradability; (iv) batch-to-batch reproducibility; (v) pharmacokinetic profile and clearance; (vi) toxicity and immunogenicity; and (vii) scalability and regulatory feasibility.

Encapsulation, therefore, represents not merely a method for enhancing physicochemical stability and biological compatibility, but a multidimensional design problem. Although numerous systems report performance improvements, success must be evaluated beyond isolated metrics, taking into account reproducibility, durability, manufacturing complexity, and regulatory feasibility. Accordingly, this section critically compares polymeric, lipid-based, inorganic, and hybrid matrices, emphasizing both performance gains and inherent trade-offs that define realistic design principles for biomedical translation.

### 3.1 Polymeric encapsulation

Polymeric encapsulation is one of the most versatile and extensively explored strategies for enhancing the biomedical performance of CDs. Through hydrogen bonding, electrostatic interactions, π–π stacking, or covalent conjugation, CDs can be integrated into nanoparticles, hydrogels, scaffolds, and nanocapsules, resulting in improved colloidal stability, controlled drug release, and fluorescence-based tracking for theranostic applications.^84^ Both natural and synthetic polymers are widely employed, each offering distinct advantages and limitations in medicinal contexts.

**Fig. 9:**
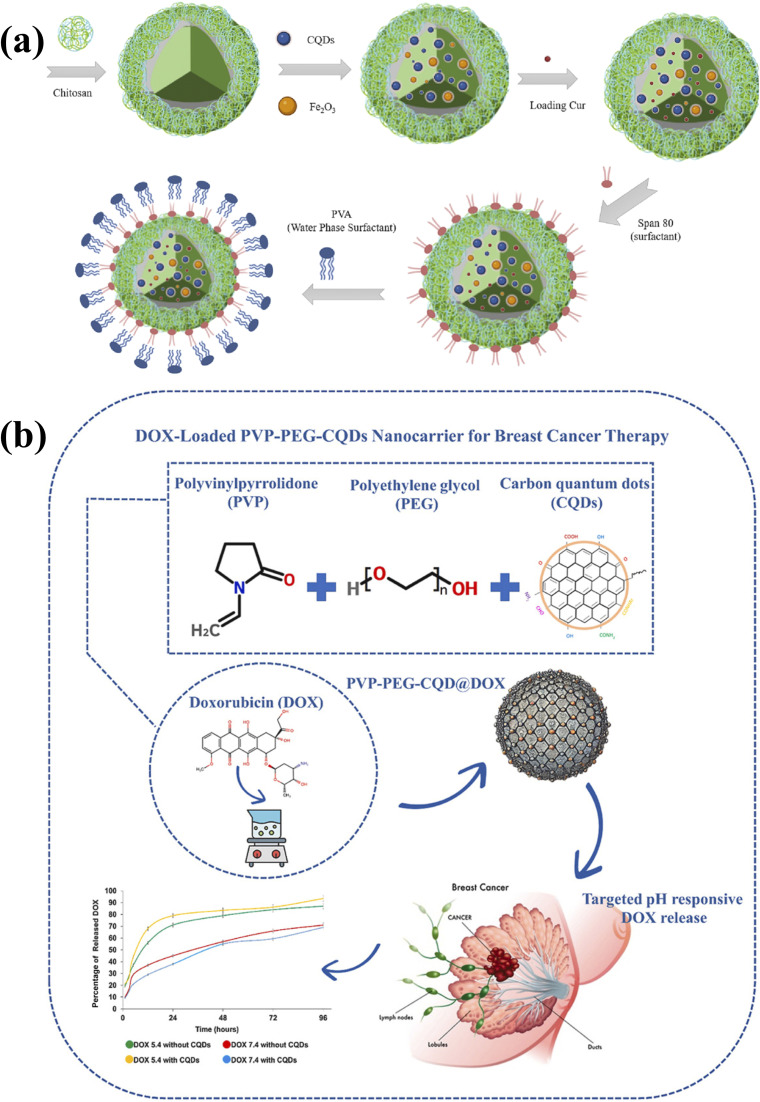
(a) Formation of curcumin-loaded CS/CQDs/Fe_2_O_3_ nanocomposite *via* W/O/W double emulsion method, reproduced from ref. 87 with permission from Elsevier,^87^ Copyright 2023. (b) Fabrication of PVP-PEG-CQDs hydrogel nanocomposite *via* double emulsion method, showing CQD integration, pH-responsive DOX release, and enhanced cytotoxicity in breast cancer cells, reproduced from ref. 79 with permission from Elsevier,^79^ Copyright 2025.

Natural polymers, including chitosan, alginate, dextran, and hyaluronic acid (HA), are particularly attractive due to their inherent biocompatibility, biodegradability, and low immunogenicity.^75,85,86^ Chitosan, a cationic polysaccharide, enhances cellular uptake *via* electrostatic interactions with negatively charged membranes and enables pH-responsive drug release under acidic tumor conditions.^87,88^ For example, a chitosan/carbon quantum dot/polyvinylpyrrolidone (CS-CQD-PVP) nanocarrier engineered for doxorubicin (DOX) delivery demonstrated high encapsulation efficiency, sustained pH-responsive release, and significantly enhanced apoptosis in breast cancer cells compared to free DOX.^89^ Similarly, curcumin-loaded CS/CQDs/Fe_2_O_3_ hydrogels fabricated *via* a W/O/W double-emulsion method (Fig. 9a) protected hydrophobic drugs from premature degradation while enabling controlled release under acidic conditions.^87^

Alginate, an anionic polysaccharide, undergoes mild ionotropic gelation with divalent cations, making it suitable for oral delivery platforms. In a chitosan/alginate hydrogel system encapsulating CD-based nanozymes for colitis therapy, the matrix effectively shielded CDs from gastric degradation and enabled targeted intestinal release, combining ROS scavenging and bioimaging capabilities.^90^ Hydrophilic polysaccharides such as dextran and hyaluronic acid (HA) further extend circulation time by imparting “stealth” properties. HA additionally enables active targeting through CD44 receptor recognition,^91^ as demonstrated by HA-CQDs in triple-negative breast cancer models, where enhanced tumor accumulation, ROS-mediated ferroptosis, and imaging capability were simultaneously achieved.^92,93^

Despite these advantages, natural polymer systems are not without limitations. Several reports note variability in solubility, sensitivity to environmental conditions, and occasional reductions in quantum yield following conjugation. Such factors may complicate reproducibility and long-term storage stability, underscoring the need for optimized modification and standardization strategies.

Synthetic polymers offer complementary advantages, particularly in terms of structural control, reproducibility, and tunable degradation profiles.^94^ Polyethylene glycol (PEG) is widely used to enhance colloidal stability, reduce opsonization, and prolong systemic circulation. A polyacrylic acid/polyethylene glycol carbon quantum dot (PAA-PEG-CQD) nanoplatform for quercetin delivery achieved 89% encapsulation efficiency and pH-responsive release, resulting in enhanced apoptosis in MCF-7 cells.^95^ PEG functionalization has also been combined with mesoporous silica-encapsulated CDs to enable simultaneous fluorescence imaging and controlled doxorubicin release.^96^

Poly(lactic-*co*-glycolic acid) (PLGA), an FDA-approved biodegradable copolymer, is particularly valued for its predictable degradation kinetics and sustained release capability. Hybrid systems incorporating silk sericin CDs into PLA/sericin nanogels within electrospun PLGA fibers demonstrated pH-dependent drug release and accelerated wound healing without cytotoxicity.^97^ Poly(ε-caprolactone) (PCL), known for slow degradation and mechanical robustness, has been used in magnesium-loaded CQD scaffolds to promote angiogenesis and wound closure.^98^ Thermo-responsive polymers such as poly(*N*-isopropylacrylamide) (PNIPAM) enable dynamic systems for selective biosensing applications.^99^ Additionally, PVP-PEG-CQD hydrogel nanocomposites fabricated *via* double emulsion (Fig. 9b) achieved high drug loading (58%) and encapsulation efficiency (92%), enabling sustained pH-responsive DOX release over 96 hours and enhanced cytotoxicity in breast cancer cells.^79^

However, synthetic polymers also present challenges. Biodegradable polyesters such as PLGA and PCL may generate acidic degradation byproducts that could induce localized inflammatory responses if not carefully formulated and purified. Furthermore, multistep fabrication routes and precise compositional control are often required to maintain batch reproducibility.

When natural and synthetic polymer systems are evaluated comparatively rather than individually, clear performance trends emerge. Although natural polymers such as chitosan and alginate offer intrinsic biocompatibility and facile functionalization, their sensitivity to environmental conditions and potential batch-to-batch variability can hinder reproducibility and scale-up. In contrast, synthetic polymers, particularly PLGA and PEG-based systems, consistently demonstrate improved structural control, predictable degradation behavior, and greater regulatory compatibility. As a result, despite extensive optimization efforts in natural polymer platforms, synthetic polymer-based systems increasingly dominate translationally oriented and clinically aligned studies.

Polymeric encapsulation is a dynamic platform that can provide improvements of colloidal stability and control release while retaining carbon dots tracking *via* fluorescence capability. Natural polymers (*e.g.*, chitosan, alginate, hyaluronic acid) are of great interest for their biodegradability and biological interaction, their environmental sensitivity, however, can limit the reproducibility (batch-to-batch differences). Synthetic polymers (notably PEG- and PLGA-based systems) have better structural control and have a better chance to scale up production, yet formulation complexity and degradation byproducts could introduce safety or manufacturing limitations. Overall, after determining the intended route of administration of the compound, the choice of polymer should be guided by the balance that needs to be struck between biodegradability, reproducibility, and translational feasibility.

### 3.2 Lipid-based encapsulation

Lipid-based nanocarriers have become one of the most adaptable and clinically promising strategies for CD encapsulation. Their intrinsic biocompatibility, structural versatility, and ability to self-assemble into bilayers, vesicles, and complex mesophases provide an effective platform for improving CD stability and biomedical functionality. By embedding CDs within lipid matrices, aggregation and poor colloidal stability can be minimized while enabling simultaneous drug delivery and imaging. This dual protective and functional role has positioned lipid-based encapsulation as a central approach in CD-based theranostics.

Liposomes constitute the most extensively studied lipid-based CD carriers. Co-loaded liposomal systems incorporating CDs and chemotherapeutic agents, most notably doxorubicin, have demonstrated multifunctional performance, combining fluorescence imaging with enhanced anticancer efficacy. These systems improve intracellular drug accumulation and modulate key signaling pathways such as PI3K/Akt/mTOR and MAPK, resulting in synergistic therapeutic responses relative to free drug administration.^100^ Beyond conventional liposomes, lipid cubosomes have emerged as structurally distinct carriers. Their bicontinuous cubic phase enhances photoluminescence intensity, improves cellular internalization, and increases bioimaging resolution, thereby expanding the structural diversity of lipid nanophases for CD delivery.^101^

**Fig. 10:**
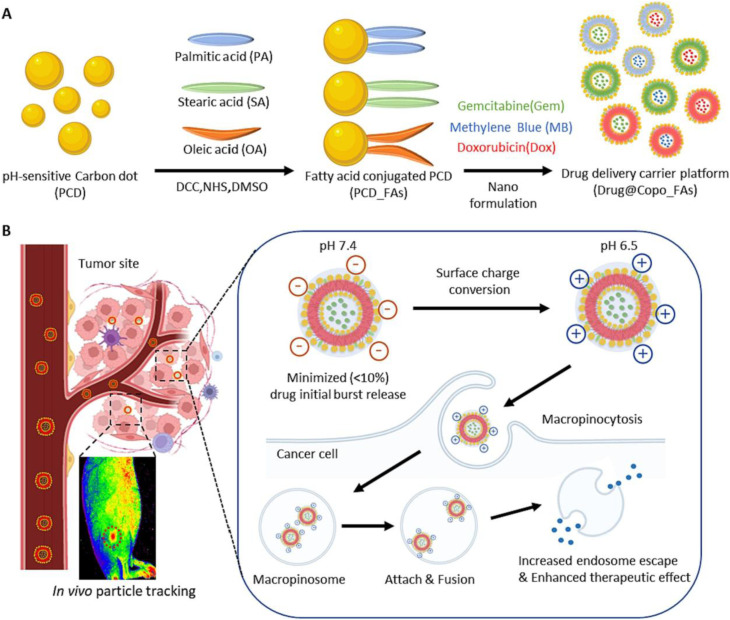
Schematic illustration of Drug@Copo_FAs nanocarriers: (A) assembly of PCD_FAs with therapeutic agents; (B) tumor-specific charge conversion, cellular uptake, and pH-responsive drug release mechanisms, reproduced from ref. 102 with permission from Elsevier,^102^ Copyright 2023.

A major conceptual advancement is the development of stimulus-responsive lipid-mimetic systems. Kim *et al.*^102^ reported amphiphilic PCD-FAs materials generated by conjugating pH-sensitive carbon dots with fatty acids. Upon drug loading, these conjugates self-assemble into vesicular “coposomes” (Drug@Copo-FAs). As illustrated in Fig. 10, the system undergoes tumor-specific surface charge conversion in acidic microenvironments, which promotes enhanced cellular uptake and subsequent vesicle destabilization to enable controlled drug release. Under physiological conditions, the nanocarriers maintain structural integrity and low leakage, while demonstrating selective cytotoxicity in tumor tissue following pH-triggered disassembly. This mechanism directly links lipid self-assembly with environmental responsiveness, representing a refined strategy for improving therapeutic precision.

Further diversification of lipid coatings has been achieved using natural product-derived lipid components to generate red fluorescent CDs with strong bioimaging capability and synergistic photothermal and photodynamic therapeutic activity against breast cancer models.^103^ In parallel, simpler lipid-coated carbon quantum dots have been shown to modulate cellular uptake behavior and cytotoxicity profiles, improving fluorescence stability while reducing nonspecific toxicity.^104^ These findings collectively demonstrate that lipid matrices can tune both biological interaction and optical performance of CDs.

Despite these advantages, lipid-based systems exhibit inherent limitations. Conventional liposomes frequently suffer from premature leakage and sensitivity to storage conditions, which can compromise long-term colloidal stability and payload retention. While cubosomes and lipid-mimetic assemblies improve structural robustness, they often require precise compositional control and multi-component assembly processes that may complicate scale-up. Moreover, although lipid coatings reduce nonspecific toxicity, their drug-loading capacity is sometimes lower than that achievable with polymeric matrices.

When lipid-based encapsulation strategies are evaluated comparatively across reported studies, recurring performance patterns emerge. Lipid systems consistently enhance cellular uptake, imaging capability, and short-term therapeutic efficacy. However, improvements in long-term durability and storage stability remain more limited, particularly for classical liposomal platforms. In contrast, more complex architectures such as pH-responsive coposomes demonstrate improved stability-release balance but introduce greater formulation complexity. These observations suggest a fundamental trade-off between biomimetic flexibility and structural robustness. Lipid-based carriers excel in membrane compatibility and rapid intracellular delivery, yet often require additional structural reinforcement or hybridization to achieve sustained stability and reproducibility.

Overall, lipid-based encapsulation provides a biologically intuitive and translationally relevant framework for CD delivery. Their strengths lie in membrane mimicry, imaging compatibility, and stimulus-responsive design, while their limitations primarily involve leakage control, storage sensitivity, and manufacturing complexity. Continued refinement, particularly through rational lipid composition engineering and integration into hybrid systems, will be essential to balance functionality with long-term stability in CD-based nanotheranostics.

Lipid-based carriers provide strong biocompatibility and efficient cellular interaction, which makes them useful for combined imaging and drug delivery. Across reported systems, lipid formulations often enhance uptake and short-term performance, especially when designed for stimulus-responsive release. However, many lipid platforms remain sensitive to storage conditions and may suffer from cargo leakage, and more advanced architectures can increase formulation complexity and reduce batch-to-batch consistency. These trade-offs suggest that lipid systems are best positioned for applications where membrane mimicry and delivery efficiency are priorities, while stability and manufacturability must be addressed through composition control or hybrid designs.

### 3.3 Inorganic matrix encapsulation

Encapsulation of CDs within inorganic matrices (IMs) has emerged as a structurally robust strategy to enhance luminescence stability, suppress nonradiative decay, and improve resistance to environmental quenching. By confining CDs within rigid host frameworks, vibrational relaxation and oxygen-induced triplet quenching can be significantly reduced, enabling long-lived phosphorescence and afterglow emission.^105^ These characteristics are particularly valuable for bioimaging and theranostic applications, where high signal-to-background contrast and minimized biological autofluorescence are essential for diagnostic sensitivity.

Among inorganic hosts, silica (SiO_2_) remains the most widely adopted platform due to its hydrophilicity, low toxicity, and compatibility with sol–gel processing. Encapsulation within silica matrices generates rigid Si–O–C interfacial interactions that immobilize surface states and extend excited-state lifetimes.^106^ For example, CNDs@SiO_2_ composites have demonstrated room-temperature phosphorescence (RTP) lifetimes of up to 1.86 s and phosphorescent quantum yields of 11.6%, enabling their use as afterglow imaging agents both *in vitro* and *in vivo*.^108^ Silica frameworks also facilitate energy-transfer engineering. In CNDs-RhB@SiO_2_ nanohybrids, Förster resonance energy transfer (FRET) between CDs and Rhodamine B produces red-shifted afterglow emission suitable for deeper tissue penetration.^107^ These examples illustrate how silica not only stabilizes emission but also provides a tunable optical microenvironment.

Zeolite frameworks represent a second class of inorganic hosts characterized by microporous crystalline confinement. Their rigid channels impose strong spatial restriction on embedded CDs, effectively suppressing vibrational dissipation and oxygen quenching, two primary limitations for long-lived emission. In a “dots-in-zeolites” strategy, CDs@zeolite composites achieved quantum yields up to 52.14% and delayed emission lifetimes of 350 ms under ambient conditions.^108^ Such confinement-enhanced optical efficiency highlights the value of structural rigidity for time-resolved imaging applications, where background suppression is critical. However, the highly crystalline and non-degradable nature of zeolite frameworks may limit post-synthetic functionalization and *in vivo* adaptability.

Metal–organic frameworks (MOFs) provide a more chemically versatile inorganic scaffold. Their hybrid organic–inorganic architecture combines porous confinement with tunable coordination chemistry, enabling fluorescence stabilization alongside stimulus-responsive behavior. MOF-encapsulated CDs have been explored in biosensing,^109^ imaging,^110^ and drug delivery.^111^ For instance, Nannuri *et al.*^111^ reported CDs embedded within ZIF-8 and functionalized with hyaluronic acid for targeted delivery of 5-fluorouracil, achieving dual pH- and temperature-responsive release with selective uptake by neuroblastoma cells and hemocompatibility toward erythrocytes.

**Fig. 11:**
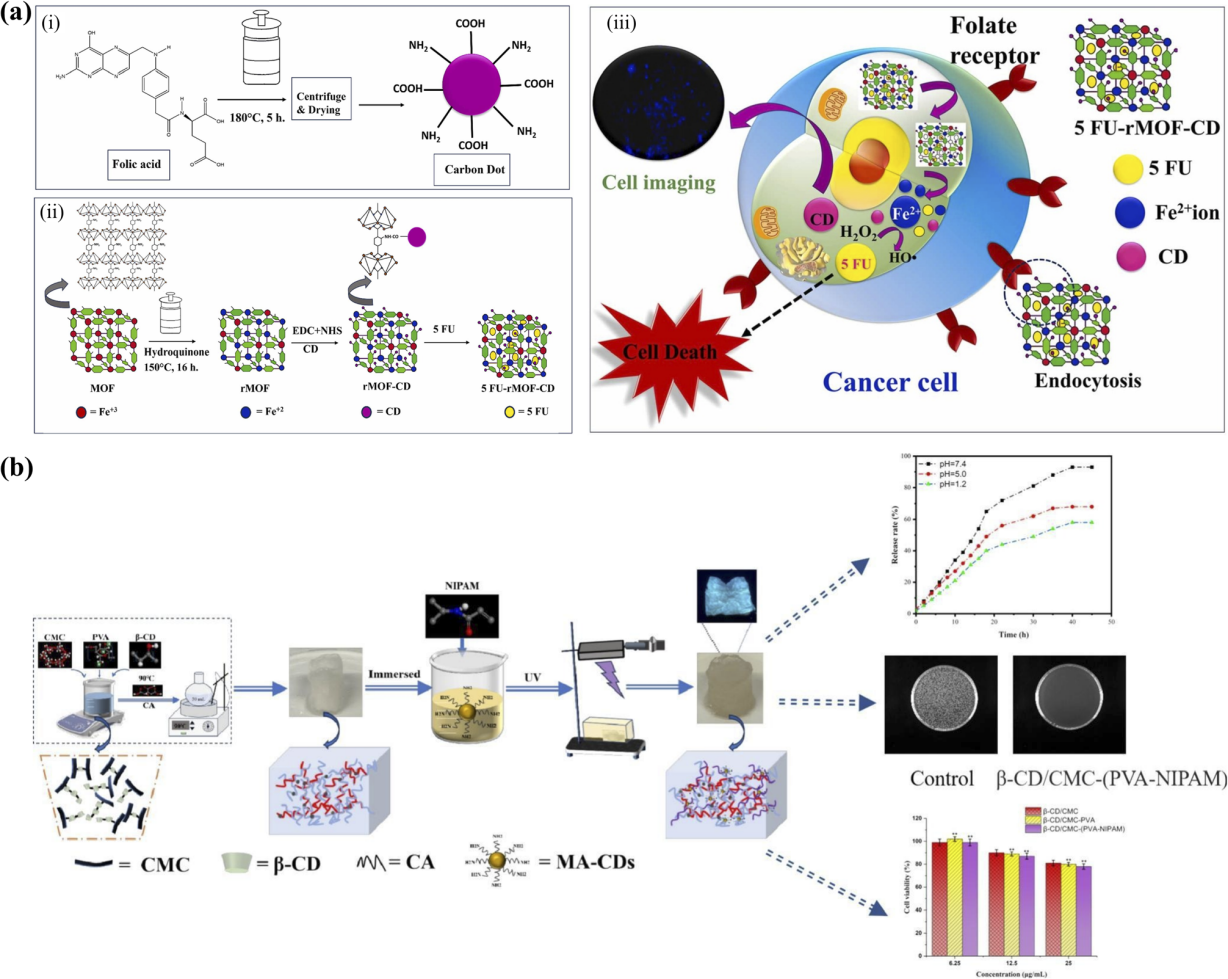
(a) Schematic of 5 FU-rMOF-CD nanoplatform for cancer theranostics: (i) synthesis of folic acid-derived CDs and reduced Fe-MOF; (ii) conjugation *via* EDC/NHS and drug loading with 5-fluorouracil; (iii) targeted delivery to folate receptor-expressing cancer cells, enabling ROS generation, chemotherapy, and fluorescence imaging, reproduced from ref. 110 with permission from Elsevier,^110^ Copyright 2024. (b) Schematic of dual stimuli-responsive β-CD/CMC-(PVA-NIPAM) hydrogel preparation and function: thermal crosslinking of β-CD/CMC/PVA followed by UV-induced polymerization with NIPAM and fluorescent CDs yields a pH- and temperature-sensitive hydrogel enabling controlled ibuprofen release, bioimaging, and biocompatibility, reproduced from ref. 82 with permission from Elsevier,^82^ Copyright 2025.

Similarly, as illustrated in Fig. 11a, Maiti *et al.*^110^ constructed a multifunctional 5-FU-rMOF-CD nanoplatform by integrating folic acid-derived CDs into a reduced Fe-MOF scaffold. Under acidic tumor conditions, scaffold destabilization facilitates Fe^2+^-mediated reactions, restoration of CD fluorescence, and controlled chemotherapeutic release. This system demonstrates how MOF matrices can simultaneously stabilize optical properties and introduce environment-triggered therapeutic activation, underscoring the theranostic potential of CDs@MOF hybrids.

When inorganic matrices are evaluated comparatively, a recurring trade-off between structural robustness and physiological adaptability becomes evident. Silica-based systems offer exceptional mechanical stability, reproducibility, and optical durability, yet their limited biodegradability may constrain long-term *in vivo* clearance. Zeolite hosts provide superior confinement and emission efficiency, but their rigid crystalline structures restrict functional integration and biological flexibility. In contrast, MOF platforms introduce chemical tunability and stimulus responsiveness; however, their coordination bonds may exhibit reduced stability under physiological conditions, potentially leading to premature degradation or variability in long-term performance.

Across these systems, inorganic encapsulation consistently enhances optical stability, prolongs emission lifetimes, and improves resistance to photobleaching relative to unconfined CDs. These advantages translate directly into improved performance in deep-tissue imaging and time-resolved biosensing. Nevertheless, structural rigidity alone does not guarantee translational suitability. Controlled degradability, physiological stability, and reproducible synthesis remain critical parameters for biomedical implementation.

Overall, inorganic matrices provide rigid confinement that can protect carbon dots from environmental quenching and improve optical stability, which is particularly valuable for high-contrast imaging. Silica and related hosts are commonly used because they are chemically robust and can be engineered into well-defined structures. At the same time, inorganic carriers may introduce concerns about long-term fate, biodegradability, and clearance, and performance can depend strongly on synthesis conditions that affect reproducibility. Therefore, inorganic encapsulation is most compelling when optical durability is the key objective, but it should be paired with clear evidence of stability in physiological media and a realistic discussion of *in vivo* clearance and safety.

### 3.4 Emerging and hybrid encapsulation strategies

Encapsulation strategies for CDs are increasingly evolving beyond single-matrix systems toward hybrid and biomimetic architectures that integrate imaging, targeting, sensing, and therapy within unified platforms.^112^ These emerging approaches aim to overcome the limitations observed in purely polymeric, lipid, or inorganic systems by combining structural stabilization with biological functionality. While these constructs demonstrate enhanced multifunctionality, their complexity introduces new considerations regarding reproducibility, scalability, and long-term stability.

Hydrogels represent one of the most actively explored hybrid platforms due to their high-water content, injectability, and responsiveness to environmental stimuli such as pH and temperature. Incorporation of CDs into hydrogel networks enables simultaneous fluorescence imaging and controlled drug release. A representative example is the dual stimuli-responsive β-CD/CMC-(PVA-NIPAM) hydrogel system (Fig. 11b), synthesized *via* thermal crosslinking followed by UV-induced polymerization with methacrylated CDs. This construct exhibits programmed ibuprofen release under intestinal pH conditions, while embedded CDs provide intrinsic fluorescence for optical tracking.^82^ Cytocompatibility and antibacterial evaluations further support its biomedical potential. However, hydrogel systems often exhibit diffusion-limited release kinetics and may suffer from structural instability under prolonged physiological exposure, particularly when mechanical robustness is required for *in vivo* applications.

Protein–CD conjugates constitute another class of hybrid systems designed to leverage intrinsic biological recognition. Human serum albumin–CD assemblies have been engineered to detect amyloid-β aggregates, inhibit fibrillization, and scavenge reactive oxygen species, thereby addressing multiple pathological features of Alzheimer's disease simultaneously.^113^ Similarly, bovine serum albumin-modified CDs have been used to encapsulate berberine, improving its aqueous solubility, mitochondrial targeting, and tumor accumulation, resulting in enhanced *in vivo* tumor suppression with reduced systemic toxicity.^114^ These protein–CD systems benefit from inherent biocompatibility and biological targeting capacity; however, protein denaturation, structural heterogeneity, and batch variability may affect long-term stability and manufacturing reproducibility.

Biomimetic cell membrane cloaking represents a further advancement in hybrid encapsulation. By coating CDs with natural cell membranes, such as macrophage or tumor-derived membranes, nanoplatforms inherit membrane proteins that confer immune evasion, prolonged circulation, and selective homing to pathological tissues. Macrophage membrane-coated CDs have demonstrated targeted delivery within inflammatory microenvironments and effective co-delivery of drugs and siRNA in osteosarcoma models.^115^ Similar membrane-coated constructs have facilitated blood–brain barrier penetration and amyloid clearance in Alzheimer's disease models.^73^ Tumor cell membrane coatings enable homotypic targeting, enhancing accumulation within cancer tissues and improving imaging-guided therapy.^116^ Mechanistic investigations further suggest that CDs can integrate into lipid bilayers and modulate membrane permeability, contributing to enhanced uptake efficiency.^117^

Despite their biological advantages, membrane-cloaked systems face challenges including membrane source variability, potential immunogenicity upon repeated administration, and complexity in isolation and purification processes. These issues highlight the trade-off between biomimetic functionality and manufacturing standardization.

Molecular imprinting introduces a distinct hybrid strategy that combines selective molecular recognition with CD photoluminescence. In molecularly imprinted polymers (MIPs), CDs are embedded within a template-directed polymer network, and removal of the template generates high-affinity recognition cavities.^118^ N,S-doped CDs integrated into silica-based MIPs have demonstrated selective detection of 4-hydroxybenzoic acid, a gastric cancer biomarker, enabling sensitive quantification in urine samples. Gelatin-based CD–MIP core–shell nanocarriers have also been developed for methotrexate delivery, achieving selective rebinding and pH-responsive drug release under tumor-like acidic conditions while minimizing premature leakage at physiological pH.^119^ Although molecular imprinting provides antibody-like selectivity and improved retention control, synthesis procedures can be multi-step and sensitive to template removal efficiency, potentially limiting scalability.

When evaluated comparatively, hybrid encapsulation systems exhibit a defining trade-off between multifunctionality and structural simplicity. Hydrogels and protein conjugates enhance biological compatibility and responsiveness but may face mechanical or stability constraints. Membrane cloaking maximizes targeting precision and immune evasion yet introduces sourcing and reproducibility challenges. Molecular imprinting achieves high specificity but often at the cost of increased fabrication complexity.

Across these diverse strategies, hybrid systems consistently expand the functional scope of CDs beyond passive stabilization toward active biological modulation. However, most remain at the proof-of-concept stage, with limited long-term *in vivo* data and insufficient standardization for large-scale production. As such, while hybrid encapsulation architectures demonstrate substantial promise for precision nanomedicine, their translational success will depend on balancing functional integration with reproducibility, degradability, and regulatory feasibility.

Hybrid encapsulation strategies combine structural engineering and biological mimicry to achieve high multifunctional integration, often bringing imaging, targeting, and stimulus-responsive therapy into a single CD-based construct. According to the standardized evaluation criteria used in this section, these systems can improve targeting efficiency and enable controlled release, but added compositional complexity may reduce batch-to-batch reproducibility and complicate scale-up and regulatory pathways. Membrane cloaking can prolong circulation and support immune evasion, yet its performance may vary with membrane source and isolation procedures. Molecular imprinting can enhance binding specificity, but it typically requires multistep synthesis and careful control of template removal, which can limit manufacturing robustness. Overall, hybrid platforms highlight a clear trade-off between multifunctionality and structural simplicity; a comparative overview of encapsulation strategies and their biomedical applications is provided in Table 1.

**Table 1:** Comparative summary of encapsulation strategies for CDs

Encapsulation strategy	Representative materials	Advantages	Limitations	Key applications	References
Natural polymer encapsulation of CDs	Chitosan, alginate, gelatin, hyaluronic acid	Biocompatible, biodegradable; pH-responsive sustained release; CD44-mediated targeting (HA); preserved fluorescence; high drug loading	Poor solubility/stability (chitosan); reduced quantum yield (HA); possible toxicity; limited *in vivo* targeting	Drug delivery (curcumin, doxorubicin, sesamol); gene/DNA delivery; bioimaging; photodynamic and ferroptosis therapy	87, 91, and 92
Synthetic polymer encapsulation	PAA/PEG hydrogels, PNIPAM-CQD microgels, PEGylated silica composites, PLGA/PCL scaffolds (*e.g.*, PCL/Mg-CQDs)	High reproducibility, tunable design, controlled drug loading/release, multifunctionality (pH-, thermo-responsiveness, imaging), structural support for regeneration	Acidic degradation products (PLGA, PCL), need for purification/surface modification, and fabrication complexity	Targeted drug delivery, biosensing, fluorescence-guided therapy, wound healing, and tissue regeneration	95, 98, and 99
Lipid-based encapsulation	Liposomes, cubosomes, lipid-tailed CDs, lipid-coated CQDs	High biocompatibility; improved stability and uptake; co-loading of drugs; stimuli-responsive release; multimodal therapy	Storage instability; batch variability; lower drug loading than polymers, complex large-scale production, and regulation	Cancer theranostics (DOX + imaging), high-resolution bioimaging, pH-sensitive tumor inhibition, photothermal/photodynamic therapy	100–103
Inorganic matrix encapsulation	Silica, zeolites, molten salts, MOFs (*e.g.*, ZIF-8)	Rigid confinement; enhanced photostability; ultralong RTP; high QY; improved dispersibility; multifunctional (drug delivery + imaging)	Synthesis complexity, limited biodegradability, and regulatory hurdles	Afterglow bioimaging, time-resolved imaging, biosensing, pH/temperature-responsive drug delivery	105, 106, 108, and 111
Hydrogel matrices	Plant-derived CD hydrogels; cellulose-based dual-network hydrogels	Injectable, stimuli-responsive; real-time imaging; high drug loading; biocompatible	Limited mechanical strength; scale-up challenges	Drug delivery, wound healing, pH sensing, bioimaging	6, 82, and 112
Protein–CD conjugates	HSA-CDs; BSA-CDs with berberine	Multi-target theranostics; ROS scavenging; improved solubility and tumor accumulation	Complex synthesis; stability concerns	Alzheimer's therapy, cancer therapy	113 and 114
Membrane cloaking	Macrophage membrane-CDs; tumor cell membrane-CDs	Immune evasion; BBB penetration; homotypic targeting; prolonged circulation	Heterogeneity of natural membranes; reproducibility issues	Alzheimer's therapy, osteosarcoma treatment, tumor targeting	73, and 115–117
Molecular imprinting (MIPs)	N,S-CDs@SiO_2_ MIPs; gelatin-based CD-MIP	Antibody-like selectivity; pH-responsive release; fluorescence sensing	Template removal, stability, and porosity control	Cancer biomarker detection, targeted drug delivery	118 and 119

### 3.5 Limitations and failure modes in carbon dot encapsulation

Although encapsulation strategies have substantially enhanced the physicochemical stability and biomedical applicability of CDs, persistent limitations remain when systems are evaluated using standardized metrics such as serum stability, biodegradability, clearance behavior, and reproducibility. To facilitate practical validation, the principal failure modes discussed in this section are systematically linked to recommended experimental evaluation strategies in Table 2, translating conceptual limitations into measurable performance criteria.

**Table 2:** Failure modes in carbon dot encapsulation and recommended experimental validation strategies

Identified failure mode	Mechanistic origin	Recommended experimental assays	Practical evaluation objective	References
Premature cargo leakage	Membrane defects, weak matrix–cargo interactions, phase instability	Serum stability assay (37 °C, 10–50% FBS), dialysis-based release study, HPLC/UV quantification	Quantify release kinetics under physiological conditions	122
Fluorescence attenuation/quenching	Aggregation-induced quenching in dense matrices; environmental oxygen effects	Quantum yield measurement, time-resolved fluorescence spectroscopy, and photostability testing	Assess preservation of intrinsic optical properties; note silica encapsulation typically enhances fluorescence	84
Structural instability in biological media	Lipid bilayer disruption, polymer swelling, aggregation	DLS over time, zeta potential monitoring, TEM after serum incubation	Evaluate colloidal stability and aggregation resistance	122
Limited biodegradability	Dense inorganic matrices, slow hydrolysis kinetics	*In vivo* degradation tracking, ICP-MS, mass loss analysis	Determine degradation profile and persistence risk	123
Delayed clearance/tissue retention	Large particle size, RES uptake, poor degradability	Long-term biodistribution study, fluorescence imaging, radiolabel tracking	Assess organ accumulation and clearance kinetics	123
Scavenger receptor-mediated immunotoxicity	Surface charge imbalance, polyamine density leading to SR-A1 recognition	Scavenger receptor inhibition assays (SR-A1 blocking), MERTK pathway analysis, and LDH release	Evaluate inflammatory potential and macrophage recognition mechanisms	121
Batch-to-batch variability	Multistep synthesis, sensitivity to processing parameters	Cross-batch DLS comparison, encapsulation efficiency reproducibility	Establish manufacturing reproducibility	84
Metal ion leakage (MOF/inorganic systems)	Coordination bond instability, framework degradation	ICP-MS metal quantification, stability testing in physiological buffer	Assess risk of ion-mediated toxicity	110 and 111
Over-functionalization effects	Excess ligand density, steric hindrance, and altered uptake pathways	Cellular uptake studies (flow cytometry), SR-blocking assay, and confocal imaging	Determine whether added functionality yields meaningful biological gain	104 and 121

One persistent challenge is encapsulation-induced fluorescence attenuation. While structural confinement within dense polymer matrices or rigid inorganic shells can suppress nonradiative decay and prolong excited-state lifetimes, excessive restriction may perturb surface-state emission or alter electronic transitions.^120^ In some systems, efforts to enhance rigidity and photostability have inadvertently reduced quantum yield or shifted emission profiles.^84^ This effect underscores a central design trade-off: the same structural features that stabilize luminescence may simultaneously compromise intrinsic emissive efficiency. Experimentally, such attenuation can be evaluated using quantum yield measurements, time-resolved fluorescence lifetime analysis, and photostability testing under physiological media conditions, as summarized in Table 2.

Over-functionalization represents another commonly overlooked limitation. Surface modification is widely employed to enhance targeting specificity, improve colloidal stability, or introduce stimulus responsiveness. Experimental studies further show that conjugation with bulky or charged ligands can reduce cellular uptake, as demonstrated in lipid-coated carbon quantum dots, where the addition of a cationic lipid layer decreased uptake in cancer cells relative to unmodified CDs.^104^ Moreover, investigations into receptor-mediated internalization reveal that surface chemistry strongly dictates recognition by scavenger receptors, indicating that functionalization can unintentionally shift uptake pathways or immunological interactions.^121^ In highly engineered multifunctional platforms, incremental improvements in targeting or responsiveness may produce diminishing therapeutic returns while increasing synthetic complexity and variability. Thus, multifunctionality does not necessarily correlate with enhanced biological performance. To further illustrate this phenomenon, representative examples across different encapsulation platforms demonstrate how incremental functional integration may yield modest performance gains while increasing formulation complexity.

Hyaluronic-acid- modified carbon dots (HA-CDS) are also capable of successful targeting of cancer cells overexpressing CD44 and exhibit enhanced uptake compared to cancer cells on CD44-negative controls, whether using imaging-guided photodynamic therapy platforms or ferroptosis-inducing HA-CQDs.^92,93^ Nevertheless, these advantages go along with structural weaknesses of ligand-functionalized nanocarriers. Densification of ligands may cause steric crowding, aggregation, and a decrease in colloidal stability, which are failure modes that directly affect the strength of encapsulation. Since HA-CDs are based on surface-retained polysaccharide fragments to target them, any slight change in surface chemistry or incomplete conjugation will cause a change in receptor affinity and batch reproducibility. Every further step of modification also implies higher chances of heterogeneous surface coverage or unintended byproducts, which makes purification and characterization difficult. Consequently, the resulting efficiency improvements in the targeting are not necessarily always worth the increased vulnerability to instability and manufacturing unpredictability.

Drug@Copo-FA lipid-mimetic coposome systems feature pH-dependent conversion of charge, intrinsic fluorescence functionality, and encapsulation of hydrophilic drugs in one amphiphilic system.^102^ The leakage of drugs is significantly low and intracellular release is better than that in conventional liposomes, but encapsulation capability is more than sensitive to the fattyacid tail structure and hydrophobic interaction that stabilizes the vesicle membrane. The change of tail length, saturation, or conjugation ability can cause changes in nanoparticle self-assembly, which results in untimely leakage, modified release kinetics, or imprecise particle size distributions. Moreover, the covalent conjugation of fatty acids to carbon dots, though synthetically simple, provides possible failure modes including incomplete esterification or heterogeneous replacement, which can damage the membrane integrity. In this way, although coposomes are multifunctional, they are susceptible to instability caused by formulation in various ways that should be properly regulated to prevent encapsulation failure.

Another set of encapsulation problems arises with hybrid MOF-carbon dot systems. Acidic tumor-like conditions can activate drug loading, fluorescence activation, and Fe^2+^-mediated Fenton activity in Fe-based rMOF-CD constructs, but also enhance MOF degradation and destabilize the coordination network.^110^ Amine linkages are protonated to weaken the rMOF-CD interface, causing it to disassemble prematurely, releasing Fe^2+^ uncontrollably, and losing its structural integrity, failure modes that directly influence drug retention and targeting accuracy. Likewise, ZIF-8@CD composites are also pH- and temperature-sensitive encapsulation systems, although the stability of the encapsulation heavily relies on the preservation of the crystalline structure and inhibition of premature collapse of pores or loss of the ligand used.^111^ Surface functionalization of composite surfaces, *e.g.*, HA coating, further aggravates the risk of heterogeneous coverage or destabilization in biological media. On these MOF-CD systems, the versatile architecture is both broadening theranostic potential and, at the same time, increasing the number of degradation routes, rendering structural stability and reproducibility the primary hurdles in clinical implementation.

These case studies highlight an important connection between the versatility of carbon-dot encapsulation and its structural fragility. Each added targeting ligand, amphiphilic component, or hybrid framework introduces potential vulnerabilities, such as surface-crowding effects or pH-driven degradation. The features that enhance functionality can also undermine reliability. Therefore, it is vital to understand these trade-offs when designing carbon-dot-based carriers to ensure a balance between multifunctionality and consistent performance in biological environments.

Encapsulation matrices impose distinct platform-specific limitations that influence the performance of carbon-dot-based systems in medicinal applications. Lipid-based carriers, while attractive for their biomimetic membrane properties and compatibility with CD–lipid hybrid platforms, are known to exhibit instability arising from membrane packing defects and phase-behavior sensitivity, which can affect cargo retention and structural integrity in biological environments. Recent analyses of CD–lipid interactions emphasize that lipid vesicles are highly responsive to changes in membrane composition and packing density, leading to variable nanoparticle insertion and perturbation of bilayer structure, factors that can compromise long-term stability in serum-containing media. These instability phenomena are typically assessed through serum leakage assays, dynamic light scattering (DLS) monitoring of size evolution, and long-term incubation studies under simulated physiological conditions (Table 2).^122^ Silica-based inorganic matrices provide robust optical confinement, high surface area, and tunable porosity, enabling efficient loading of CDs and other therapeutic agents. Nonetheless, CD-silica hybrid nanostructures display the slow and environment-sensitive degradation typical of silica, which may complicate biological elimination and trigger concerns about prolonged tissue retention, especially in the case of dense or non-porous silica structures.^123^ Biodegradation and clearance limitations in such systems can be quantified using *in vivo* degradation tracking, elemental analysis of tissue retention, and longitudinal biodistribution profiling, as outlined in Table 2. Hybrid systems that combine lipid, polymer, or inorganic components provide multiple functions and better control over their optical and structural features. However, these systems often need complicated, multi-step synthesis methods like sol–gel processing, inverse microemulsion templating, or molecular imprinting. These methods can create difficulties with reproducibility, scaling up for larger production, and meeting regulatory standards because of their complex structures and sensitivity to processing conditions.^123^

Beyond material-specific issues, scalability and reproducibility remain major translational bottlenecks. Many encapsulation approaches rely on multistep synthesis routes, solvent-sensitive self-assembly processes, or post-synthetic functionalization that are difficult to standardize under good manufacturing practice (GMP) conditions. Precise control over particle size distribution, surface chemistry, encapsulation efficiency, and long-term stability across production batches remains challenging, particularly for complex core–shell, membrane-coated, or stimuli-responsive architectures. Batch reproducibility may be evaluated through statistical comparison of particle size distribution, encapsulation efficiency, surface charge, and optical properties across independent production runs, thereby establishing quantitative reproducibility benchmarks (Table 2).

Importantly, improvements in one performance parameter frequently impose constraints on another. Increasing structural rigidity enhances photostability but may reduce degradability and slow biological clearance. Extensive surface functionalization improves targeting but can compromise reproducibility and introduce steric barriers that diminish bioactivity. Hybridization expands multifunctionality but often increases fabrication complexity and regulatory burden. These interdependent effects demonstrate that encapsulation design is not a linear optimization problem, but a multidimensional balancing process involving stability, biodegradability, optical performance, manufacturability, and clinical feasibility.

Collectively, these limitations highlight that performance optimization alone is insufficient for successful translation. Progress in CD encapsulation will require standardized evaluation protocols incorporating long-term stability studies, degradation profiling, reproducibility assessment, and clinically relevant pharmacokinetic analysis. Only through such integrated validation can encapsulation strategies move beyond proof-of-concept demonstrations toward reliable and regulatory-aligned biomedical implementation.

## 4 Applications of encapsulated CDs in medicinal chemistry

**Fig. 12:**
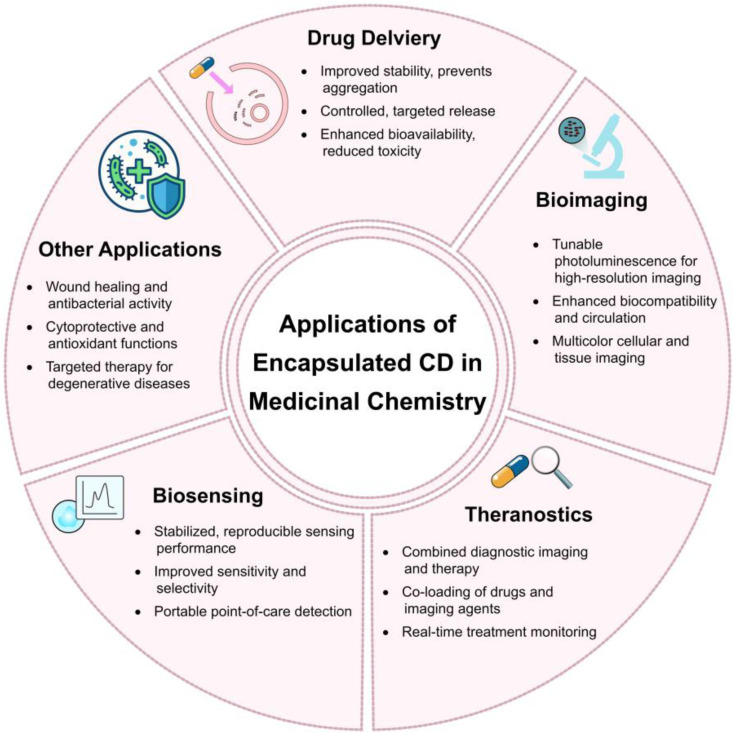
Summary of multifunctional applications for encapsulated CDs.

Encapsulated CDs have attracted significant attention in medicinal chemistry due to their excellent biocompatibility, tunable photoluminescence, and versatile surface chemistry, with encapsulation enhancing their stability, targeting ability, and functionality. These hybrid nanostructures exhibit multifunctional applications across drug delivery, bioimaging, theranostics, biosensing, and other biomedical fields. Fig. 12 presents a summary of these applications.

### 4.1 Drug delivery

The evolution of nanomedicine toward precision therapeutics has positioned encapsulated CDs as a cornerstone of advanced drug delivery platforms. By integrating CDs into polymeric, lipidic, or inorganic matrices, these hybrid systems synergize the inherent biocompatibility, tunable surface chemistry, and intrinsic fluorescence of CDs with the protective and functional capabilities of the encapsulating material.^124,125^ This convergence enables unprecedented control over drug loading, targeted delivery, and stimuli-responsive release, while facilitating real-time tracking of biodistribution and therapeutic efficacy, a hallmark of theranostic nanomedicine.

Drug incorporation into CD-based nanocarriers can occur through several mechanisms, each influencing loading efficiency, release kinetics, and targeting specificity. Covalent attachment leverages abundant surface functional groups, such as amine, carboxyl, or hydroxyl moieties, to form stable chemical linkages with therapeutic agents. Nitrogen-doped CDs exemplify this strategy by forming strong covalent bonds with doxorubicin (DOX), resulting in pH-responsive release and sustained therapeutic activity within acidic tumor microenvironments.^126^ In addition to covalent conjugation, non-covalent mechanisms such as hydrogen bonding, π–π stacking, and electrostatic interactions facilitate reversible drug loading, enabling environmentally sensitive release profiles. Physical entrapment within polymeric hydrogels, lipid bilayers, or inorganic matrices further enhances loading capacity and supports prolonged release. Hydrogel-based systems, for instance, utilize the porous network of polymer matrices to achieve high drug entrapment efficiency and stable, long-term bioavailability.^68,95^

The therapeutic advantages of encapsulated CDs extend beyond drug loading due to their intrinsic photoluminescence, which supports real-time visualization of nanocarrier biodistribution and therapeutic progression. Porphyrin-derived CDs loaded with DOX have demonstrated exceptionally high drug-loading efficiencies (up to 93%) and have enabled fluorescence-guided chemotherapy, significantly outperforming free DOX in cytotoxicity assays.^78^ Stimuli-responsive behavior further enhances therapeutic precision. For example, PVP-PEG-CQD hydrogels exhibit selective DOX release under acidic conditions, leading to enhanced apoptosis in breast cancer models relative to free DOX treatments.^79^ Likewise, gelatin/PEG/Cu-doped CQDs encapsulating curcumin exhibited pH-sensitive release and selective cytotoxicity against glioblastoma cells, while maintaining >90% viability in healthy fibroblasts.^68^

**Fig. 13:**
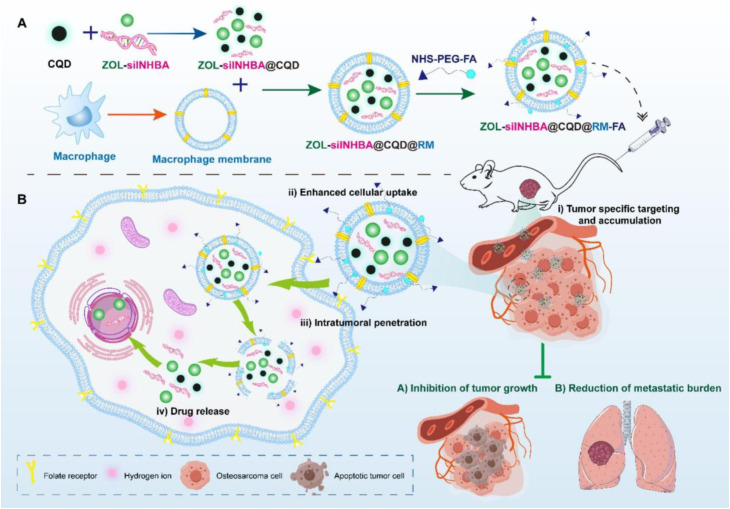
(A) Preparation of the folic acid-modified macrophage membrane-coated carbon quantum dot nanoplatform (ZOL-siINHBA@CQD@RM-FA), showing co-encapsulation of zoledronic acid and INHBA siRNA. (B) Schematic mechanism of tumor-specific internalization, pH-responsive release, and synergistic suppression of osteosarcoma growth and metastasis, reproduced from ref. 115 with permission from Elsevier,^115^ Copyright 2025.

Targeting capabilities are further enhanced through surface functionalization and biomimetic strategies. Yao *et al.*^115^ developed a folic acid-modified macrophage membrane-coated CQD nanoplatform (ZOL-siINHBA@CQD@RM-FA) for the co-delivery of zoledronic acid (ZOL) and Inhibin Subunit Beta A (INHBA) siRNA, representing a significant advancement in biomimetic dual drug and gene delivery for osteosarcoma therapy. This nanoplatform enabled efficient co-delivery of therapeutic agents, exhibited pH-responsive release, demonstrated selective tumor internalization, and showed high biosafety. Intravenous administration suppressed osteosarcoma proliferation, reduced orthotopic tumor burden, and inhibited lung metastasis without systemic toxicity. The schematic in Fig. 13 illustrates the formulation process and mechanism of action, highlighting how CQDs encapsulated within folic acid-modified macrophage membranes achieve precise tumor targeting, controlled release, and synergistic therapeutic effects. This biomimetic design underscores the potential of CD-based nanocarriers to integrate chemotherapeutic and genetic therapies, expanding the therapeutic scope of encapsulated carbon dot systems in advanced cancer management.

Further functionalization has been achieved by integrating inorganic components to create multifunctional systems for combination therapies. For example, the incorporation of Fe_2_O_3_ or lanthanum doping imparts magnetic properties, enabling magnetic field-guided drug delivery, enhanced imaging contrast, and improved therapeutic stability.^87,127^ These multifunctional designs highlight how CDs can be engineered not only as passive carriers but as active participants in targeted therapy, integrating diagnostic and therapeutic functions. Beyond cancer, CD-based encapsulation has been extended to antimicrobial and wound-healing applications. Antibiotic-conjugated CDs derived from biomass demonstrated enhanced antibacterial activity against resistant strains,^128^ while PHMG-derived CDs incorporated into hydrogels provided sustained release, broad-spectrum antibacterial activity, and immunomodulatory effects for infected wound healing.^129^ These findings show the versatility of CDs in addressing both oncological and infectious disease challenges.

Overall, encapsulated CD systems offer high drug-loading efficiency, tunable and stimuli-responsive release, reduced systemic toxicity, and improved therapeutic outcomes across diverse biomedical contexts. Although issues such as production scalability, long-term biosafety, and regulatory approval remain obstacles to clinical translation, ongoing advancements in polymeric, lipid-based, inorganic, and biomimetic encapsulation methods continue to expand the potential of CDs as next-generation nanocarriers for precision drug delivery. Table 3 summarizes recent studies on the application of encapsulated CDs in drug delivery.

**Table 3:** Carbon dot modifications, associated therapeutic agents, delivery matrices, and their observed outcomes in drug delivery applications

Carbon dot type/modification	Therapeutic agent	Delivery method/matrix	Key outcomes	References
Gelatin/polyethylene glycol/copper-doped carbon quantum dots (G/PEG/Cu.CQDs)	Curcumin	Gelatin/PEG nanocarrier (W/O/W emulsion)	97% release at pH 5.4; selective glioblastoma cytotoxicity; >90% healthy cell viability	68
Polyvinylpyrrolidone/carbon quantum dots/polyethylene glycol (PVP-CQDs-PEG)	Doxorubicin	Hydrogel nanocomposite	92% encapsulation; pH-responsive release; enhanced apoptosis in MCF-7 cells	79
Gelatin/agarose/magnesium-doped carbon quantum dots (Gel/Aga/Mg-CQDs)	Curcumin	Gelatin/agarose hydrogel	85.5% encapsulation; sustained acidic release; selective glioma toxicity	81
Carbon quantum dots embedded in dual stimuli-responsive cellulose-based hydrogel (C-CDs@DN hydrogel)	Ibuprofen	Dual-network hydrogel (β-CD/CMC/PVA-NIPAM)	96% release; pH and temperature-responsive; low cytotoxicity	82
Porphyrin-derived carbon dots (PCDs)	Doxorubicin	Non-covalent loading	93% loading; pH-responsive release; improved cytotoxicity in breast cancer cells	78
Chitosan/carbon quantum dots/Fe_2_O_3_ nanocomposite (CS/CQDs/Fe_2_O_3_)	Curcumin	Chitosan-based nanocomposite	Controlled release; enhanced stability; selective cytotoxicity in MCF-7 cells	87
Polyacrylic acid/polyethylene glycol/carbon quantum dots (PAA-PEG-CQDs)	Quercetin	Hydrogel nanoplatform	89% encapsulation; sustained acidic release; increased apoptosis	95
Bovine serum albumin-modified carbon dots (BSA-CDs)	Berberine	Protein-CD composite	Enhanced solubility; mitochondrial targeting; tumor suppression *in vivo*	114
Folic acid-targeted macrophage membrane-coated carbon quantum dots (RM-FA-CQDs)	Zoledronic acid + siINHBA	Macrophage membrane biomimetic nanoplatform	Dual delivery; pH-responsive release; metastasis inhibition	115
Nitrogen-doped carbon dots (N-CDs)	Doxorubicin	Covalent Schiff base linkage	35.88% loading; sustained release; two-photon imaging	126
Lanthanum-doped carbon dots (La-CDs)	Camptothecin	β-CD/PEG/folate composite	>90% encapsulation; sustained release; cell cycle arrest	127
Carbon dots embedded metal organic framework@chitosan (CDs@MOF@OCMC)	Doxorubicin	Core–shell MOF with chitosan	Dual-mode imaging (FOI/MRI); pH-triggered release	26
Gelatin-based carbon quantum dot molecularly imprinted polymer (g-CQD-MIP)	Methotrexate	Gelatin-based molecularly imprinted polymer	pH-responsive release; selective cytotoxicity; imprinting factor 4.91	119
Carbon dot-metal organic gel nanocarrier (CD-MOG)	5-Fluorouracil	Metal–organic gel	72.9% loading; light-triggered release; chemo-photodynamic therapy	130
Graphene oxide-assisted N,S co-doped carbon quantum dots (GO@N,S-CQDs)	Letrozole	Thermosensitive polymer	90% release in 15 min under NIR; zero-order kinetics	131
Wild *Citrullus lanatus*-derived carbon dots (CL-CDs)	Ampicillin sodium	Conjugated CDs	Enhanced antibacterial activity; inhibition of resistant strains	128
Polyhexamethylene guanidine-derived carbon dots (PHMG-CDs)	Antibacterial hydrogel	ε-PLL/chitosan hydrogel	Sustained release; antibacterial + immunomodulatory; wound healing	129

### 4.2 Bioimaging

Encapsulation has become a critical strategy for enhancing the performance of CDs in bioimaging applications, particularly by improving their stability, optical durability, biocompatibility, and targeting precision. Although CDs inherently possess desirable photoluminescence and biocompatibility, unencapsulated CDs often suffer from photobleaching, limited emission lifetimes, and susceptibility to quenching in complex biological environments. Encapsulation within silica, lipid matrices, cyclodextrin frameworks, or mesoporous carriers has proven effective in mitigating these limitations, enabling more reliable fluorescence and phosphorescence signals for *in vitro* and *in vivo* imaging.^132–134^ For example, silica confinement of polymer-derived CDs has yielded ultralong room-temperature phosphorescence in aqueous environments, permitting autofluorescence-free imaging with phosphorescence lifetimes extending up to 2.19 seconds, significantly surpassing the capabilities of conventional CD probes.^135^ Similarly, the covalent hybridization of CDs within dendritic mesoporous silica nanobeads has produced robust time-gated phosphorescence imaging, and folic acid functionalization further enhances the targeted visualization of folate receptor-overexpressing cancer cells.^136^

Encapsulation also facilitates targeted and subcellular imaging when CDs are functionalized with biomolecules that recognize specific cellular receptors or organelles. Hyaluronan-conjugated CDs, for instance, selectively bind to CD44 receptors, enabling high-contrast tumor imaging due to elevated CD44 expression in many malignancies.^91^ Subcellular targeting has been demonstrated using Golgi-localizing red-emissive CDs synthesized from Nile blue precursors and sulfonamide ligands, which achieve precise localization within the Golgi apparatus and emit at 645 nm, effectively bypassing autofluorescence from surrounding cellular structures.^137^ Lipid-coated CQDs derived from mango leaves show another advantage of encapsulation: their modified surface properties facilitate differential uptake between cancerous and non-cancerous cells, illustrating how encapsulation can modulate cellular interaction pathways and enhance selective imaging performance.^104^

Hybrid nanoplatforms further expand the utility of CDs by enabling multimodal imaging approaches. Gold nanoparticle-decorated phytocapped CQDs prepared *via* a green synthetic route from *Physalis minima* displayed enhanced blue fluorescence, greater photostability, and improved cellular uptake compared to unmodified CQDs.^138^ This AuNP–CQD hybrid enabled clear fluorescence imaging in both plant and HeLa cells, demonstrating how inorganic–organic hybridization elevates imaging quality across diverse biological systems. Cyclodextrin-modified CDs have also proven effective in high-contrast intracellular imaging, as their encapsulation improves aqueous compatibility, supports efficient cell penetration, and preserves the intrinsic fluorescence of the CDs.^139^ Likewise, aptamer-functionalized mesoporous silica-CD composites have demonstrated selective internalization in breast cancer cells, resulting in bright and well-defined fluorescence signals suitable for diagnostic visualization.^140^ Overall, these encapsulated systems illustrate how tailoring the chemical and structural environment around CDs enhances their selectivity, stability, and imaging accuracy.

**Fig. 14:**
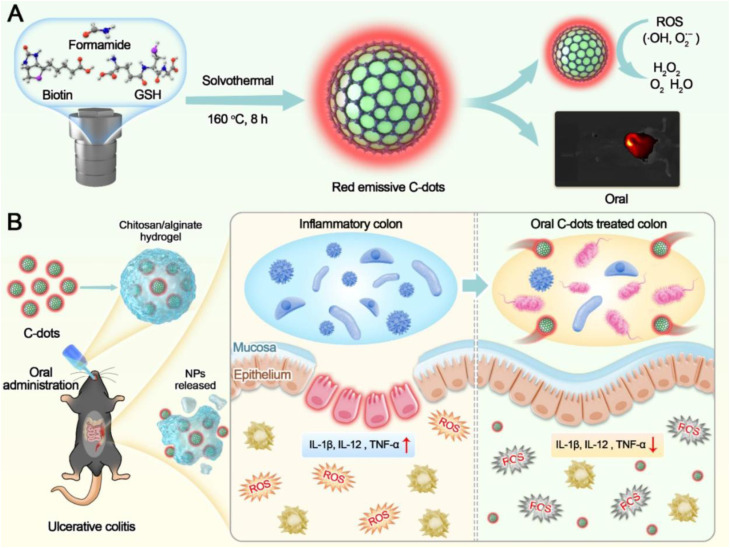
Schematic representation of glutathione/biotin-derived carbon dot nanozymes: (A) synthesis and properties for ROS scavenging and bioimaging; (B) oral hydrogel delivery to inflamed colon, enabling targeted release, red fluorescence imaging, and therapeutic modulation of ulcerative colitis, reproduced from ref. 90 with permission from Elsevier,^90^ Copyright 2024.

Encapsulation strategies are increasingly being integrated with therapeutic functionalities, advancing CDs toward multifunctional platforms capable of simultaneous diagnosis and treatment. Zhang *et al.*^90^ provide an illustrative example with glutathione/biotin-derived carbon dot nanozymes developed for both bioimaging (Fig. 14A) and management of ulcerative colitis (Fig. 14B). These nanozymes were embedded within chitosan/alginate hydrogels for oral administration, enabling targeted release at inflamed colonic sites due to pH-responsive hydrogel degradation. Upon release, the CDs exhibited red fluorescence suitable for intestinal bioimaging while simultaneously exerting reactive oxygen species (ROS) scavenging activity and modulating gut microbiota to alleviate inflammation. This example highlights the potential of encapsulated CDs not only as high-performance imaging agents but also as theranostic tools capable of integrating real-time visualization with therapeutic functions.

Overall, encapsulation significantly enhances the photophysical behavior, stability, targeting specificity, and functional versatility of CDs, enabling their application across a broad spectrum of bioimaging contexts, from subcellular labeling to inflammation tracking and multimodal diagnostic platforms. Representative encapsulated CD systems, their matrices, imaging modes, and key outcomes are summarized in Table 4.

**Table 4:** Encapsulated CDs and their modifications, encapsulation matrices, bioimaging applications, and key outcomes

Carbon dot type/modification	Encapsulation/matrix	Bioimaging application	Key outcomes	References
Polymer CDs confined in silica nanospheres (PCDs-SNSs)	Silica confinement	Autofluorescence-free imaging	Ultralong phosphorescence lifetime (2.19 s); stable in aqueous medium	135
CDs@DMSNs (covalently hybridized)	Mesoporous silica nanobeads	Time-gated phosphorescence imaging	Lifetime 1.195 s; folic acid targeting; *in vivo* imaging	136
β/γ-Cyclodextrin-modified CDs (β/γ-Cyd-CDs)	Cyclodextrin surface modification	Cell imaging	Non-cytotoxic; high cell penetration; intrinsic fluorescence	139
Golgi-targeting red-emissive CDs (RGCDs)	Nile blue-derived CDs with sulfonamide ligand	Organelle imaging	Red emission (645 nm); Golgi targeting; Pearson correlation 0.87	137
Lipid-coated mango leaf CQDs (mQDs-DOTMA)	Lipid encapsulation	Cancer *vs.* normal cell imaging	Differential uptake; reduced toxicity; proliferative effect in normal cells	104
Hyaluronan-conjugated nCQDs (HA-nCQDs)	HA functionalization	Tumor bioimaging	CD44 receptor targeting: high tumor specificity *in vivo*	91
MSNs-CDs functionalized with anti-MUC1 aptamer	Mesoporous silica encapsulation	Cancer theranostics	Selective cytotoxicity in MCF-7/MDA-MB-231; fluorescence imaging	140
AuNPs-decorated phytocapped CQDs (CQDs@AuNPs)	Green synthesis hybrid	Plant and HeLa cell imaging	Strong blue emission; sustainable synthesis; dual imaging	138
CQDs encapsulated in DMPC liposomes	Electrochemical one-pot synthesis	Optical microscopy imaging	Blue fluorescence; stable encapsulation; suitable for epifluorescence	141
Oral red-fluorescent CD nanozymes	Chitosan/alginate hydrogel	Colitis bioimaging	ROS scavenging; intestinal accumulation; inflammation imaging	90
CDs in mesoporous silica nanoparticles (MSCDs)	FA-CβCD gatekeeper	Cancer imaging + drug delivery	Targeted uptake in FA-positive cells; intrinsic fluorescence for imaging	142

### 4.3 Theranostics

The integration of CDs into encapsulated delivery systems has emerged as a powerful approach for unifying therapeutic and diagnostic functionalities within a single nanoplatform. Findings across multiple studies consistently demonstrate that encapsulation enhances the physicochemical stability of CDs, improves their biodistribution, and amplifies their performance in biological environments.^7,100,143,144^ Liposomal systems provide a clear example of this advantage; they enable the co-loading of CDs with therapeutic agents such as curcumin or doxorubicin, shielding the payload from premature degradation while simultaneously enabling fluorescence-guided tracking of drug distribution and tumor accumulation.^100,145^ One of the most notable strengths of encapsulated CDs is their versatility in supporting multiple therapy modes. Iron-doped CD hybrids, for instance, integrate tetramodal imaging with photothermal and chemodynamic therapy, highlighting the capacity of CD-based systems to unify diagnostic and therapeutic features in a single construct.^146^ Other platforms, such as hyaluronic acid-conjugated CD vesicles, take advantage of receptor-mediated uptake in lung adenocarcinoma cells, thereby enabling tumor-selective metabolic inhibition alongside fluorescence imaging.^147^ Natural product-derived CDs, such as curcumin-based nanodots, further expand this versatility by contributing intrinsic anticancer activity in addition to strong fluorescence, enabling concurrent imaging and tumor suppression.^148^

The choice of encapsulation matrix plays a determining role in shaping therapeutic release kinetics, targeting efficiency, and imaging performance. Liposomes, protein carriers, and mesoporous silica nanoparticles each bring distinct advantages that influence CD-based theranostic behavior. Hollow mesoporous silica nanoparticles co-loaded with CDs and doxorubicin represent a sophisticated design in which pH and glutathione act as dual triggers to induce controlled drug release while enabling real-time imaging through near-infrared emissive CDs.^149^ Similarly, ligand-decorated zein nanoparticles loaded with paclitaxel and carbon quantum dots achieve sustained drug release while maintaining the structural integrity of the carrier proteins, with built-in fluorescence signals providing ongoing theranostic feedback.^150^ Emerging platforms such as near-infrared CD-metal–organic framework (MOF) assemblies broaden the functionality of encapsulated CDs even further. These hybrid systems respond dynamically to tumor microenvironment stimuli, enabling real-time fluorescence imaging while activating chemodynamic and photothermal therapeutic pathways.^151^ Redox-responsive CD nanoclusters coated with cancer cell membranes offer another layer of sophistication by acting as “cluster bombs,” these constructs disassemble within reductive tumor conditions to release doxorubicin and restore fluorescence, enabling homologous targeting and tumor-specific therapy.^152^ Other complex nanoassemblies integrate natural therapeutic molecules, CDs, and metal ions to create three-way synergistic treatments. For example, hybrid constructs combining CDs, copper ions, and ursolic acid deliver chemotherapy, photodynamic therapy, and chemodynamic therapy simultaneously under tumor-specific conditions.^153^ Likewise, copper/carbon quantum dot-crosslinked nanosheets expand the diagnostic capabilities of CD systems by supporting fluorescence, photoacoustic, and photothermal imaging with additional nuclear-targeted photothermal therapeutic effects.^154^

**Fig. 15:**
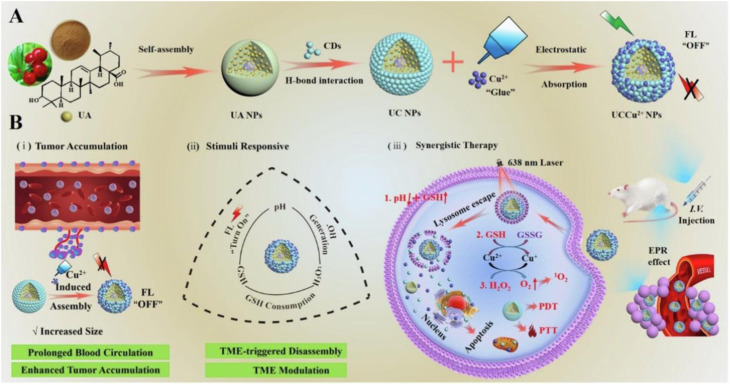
(A) Illustration of the synthesis pathway for UCCu^2+^ nanoparticles and (B) their functional features, including enhanced tumor accumulation, responsiveness to TME stimuli, lysosomal escape, and synergistic therapeutic effects, reproduced from ref. 153 with permission from Elsevier,^153^ Copyright 2023.

A detailed example of an encapsulated CD-based theranostic system is provided by Lai *et al.*,^153^ who developed hybrid nanoassemblies composed of ursolic acid (UA) nanoparticles, CDs, and Cu^2+^ ions (UCCu^2+^ NPs) for hepatocellular carcinoma therapy. As depicted in Fig. 15, the CDs within these assemblies exhibit quenched fluorescence under physiological conditions but regain emission in the acidic and reductive tumor microenvironment, thereby offering selective imaging capabilities. Coordination with Cu^2+^ not only contributes to this fluorescence modulation but also facilitates charge reversal and lysosomal escape, enhancing intracellular accumulation. In addition, tumor-associated stimuli such as low pH, elevated glutathione levels, and hydrogen peroxide promote nanoparticle disassembly, improving tumor penetration and accelerating drug release. This design results in a synergistic therapeutic platform wherein ursolic acid provides chemotherapeutic activity, CDs enable photodynamic therapy, and Cu^2+^ drives chemodynamic reactions for reactive oxygen species generation, together producing a potent, multi-mechanistic antitumor response.

Encapsulation strategies have also been leveraged to overcome drug resistance and enhance deep tumor penetration. Dendrimer/CD nanohybrids, for example, can carry dual drug payloads to bypass multidrug resistance mechanisms while maintaining fluorescence properties for imaging-guided therapy.^155^ Ultrasound-responsive dendrimer/CD constructs further improve therapeutic penetration in resistant tumors, offering enhanced imaging and drug delivery capabilities under ultrasound stimulation.^156^ Additional strategies involve integrating CDs into immunoinducible hydrogels, where photothermal therapy synergizes with immunomodulation to promote strong systemic antitumor immunity.^157^ Zeolitic imidazolate framework (ZIF)-based CD hybrids also demonstrate hierarchical size and charge transitions that enable deep tumor infiltration, with embedded CDs providing Golgi-targeted imaging while doxorubicin delivers effective chemotherapy.^158^ Recent studies compiling the theranostic applications of embedded CD systems are summarized in Table 5.

**Table 5:** Overview of encapsulated CDs, their encapsulation strategies, theranostic functions, and key outcomes

Encapsulated CDs	Encapsulation strategy	Theranostic function	Key outcome	References
Boron-doped CQDs	Biocompatible nanoplatform	Imaging + selective cytotoxicity	High quantum yield; organ-specific biodistribution; selective cytotoxicity	159
Herbal extract-derived N/S co-doped CDs with thymol and menthol	Chitosan encapsulation	Anticancer activity + fluorescence imaging	Sustained release; biocompatibility; cytotoxicity against MCF-7 cells	74
CD-based nanozyme	DOX/siRNA cocktail encapsulation	Chemo + gene therapy + imaging	Acid-triggered release; ROS generation; reversal of drug resistance	160
CDs-PEG-FA conjugates	Folate-targeted PEGylated CDs	NIR-triggered DOX release + imaging	50% enhanced release under NIR; receptor-mediated uptake	161
CD44-targeted liposomes with curcumin and CDs	Liposomal encapsulation	Fluorescence imaging + radiosensitization	Dual encapsulation; enhanced uptake; improved imaging contrast	145
CDs-NHF liposomes with DOX	Dual-functional liposomal nanoplatform	Chemo + imaging; pathway inhibition	Downregulation of pAKT/pmTOR/pERK; enhanced cytotoxicity	100
Dual targeting of CD44 and SLC1A5 in lung adenocarcinoma	HA-CD vesicle conjugates with DON	Metabolic inhibition + imaging	6-Fold increase in cytotoxicity; selective uptake in A549 cells	147
Curcumin-derived CQDs	Bottom-up synthesis from *Curcuma long*a	Fluorescence imaging + apoptosis induction	Strong fluorescence; tumor suppression *in vivo*	148
Dual-stimuli responsive NIR CDs	CDs/HMSN with PLL(cit) coating	pH/GSH-triggered DOX release + NIR imaging	Fluorescence recovery; reduced side effects; real-time imaging	149
CDs embedded in chitosan/gelatin	Crosslinked polymer matrix	Fluorescence imaging + controlled drug release	Stable luminescence; dopamine release; biocompatibility	75
Paclitaxel and CQDs co-loaded in zein nanoparticles	Ligand-decorated protein carrier	Sustained release + fluorescence imaging	>80% drug entrapment; biphasic release; retained protein structure	150
NIR CD-MOF assemblies for TME-activated therapy	MIL-100(Fe) MOF hybrid	Imaging + chemodynamic + photothermal therapy	GSH-responsive fluorescence; Fe^2+^ release; synergistic tumor inhibition	151
Redox-responsive CD nanoclusters coated with cancer cell membranes	DOX-loaded CD clusters	Homologous targeting + imaging	Fluorescence turn-on in TME; immune escape; enhanced penetration	152
Hybrid nanoassemblies of natural product, CDs, and Cu^2+^	Self-assembled nanoplatform	Synergistic HCC therapy (chemo + PDT + CDT)	TME-responsive disassembly; ROS generation; lysosomal escape	153
Copper/CD-crosslinked nanosheets	PEGylated nanosheets	Fluorescence + photoacoustic + photothermal imaging	41.3% photothermal conversion; nuclear targeting; multimodal imaging	154
Dual drug-loaded dendrimer/CD nanohybrids	PAMAM dendrimer with DOX + TPGS	MDR reversal + imaging	P-gp inhibition; targeted delivery; fluorescence tracking	155
Ultrasound-enhanced dendrimer/CD nanohybrids	UTMD-assisted delivery	MDR tumor therapy + imaging	pH-triggered release; ultrasound-enhanced uptake; improved efficacy	156
Immunoinducible CD-incorporated hydrogels	Mannose/Al-CD hydrogel depot	Photothermal therapy + immune activation	CpG ODN delivery; dendritic cell maturation; robust immune response	157
ZIF-8-derived CDs	Hierarchical size/charge transformation	Deep tumor penetration + fluorescence imaging	Golgi-mediated transcytosis; enhanced DOX delivery; imaging	158

Taken together, these findings highlight the extraordinary breadth of theranostic opportunities enabled by encapsulated CDs. These nanomaterials function simultaneously as therapeutic carriers, imaging agents, and environment-responsive probes capable of navigating the complexities of tumor biology. The path ahead now lies in translating these advances into clinically viable nanomedicines, where reproducible synthesis, long-term biosafety, and scalable manufacturing will be critical determinants of clinical impact.

### 4.4 Biosensing

Encapsulation has become a central strategy for enhancing the biosensing capabilities of CDs, particularly in applications requiring stability, selectivity, and reliable performance in complex biological environments. A growing body of research (Table 6) demonstrates that encapsulation preserves luminescence, minimizes quenching, and improves analytical accuracy. For instance, silica nanospheres containing grapefruit peel-derived CQDs have been used to develop a photoluminescence immunoassay for detecting the tumor suppressor protein p53 in serum with high sensitivity and significantly reduced interference relative to traditional immunoassays.^162^ Encapsulation within porous architectures such as mesoporous silica or metal–organic frameworks (MOFs) offers further advantages by enhancing fluorescence output and suppressing external quenchers. Wang *et al.*^163^ developed a ZIF-8 co-encapsulation system combining CDs and gold nanoclusters, which provided acid-responsive dual-emission sensing for urease activity in saliva. This platform not only achieved accurate biochemical analysis but also incorporated a smartphone-based readout, demonstrating how encapsulation can facilitate practical and portable biosensing formats.

**Table 6:** Summary of encapsulated CDs, their encapsulation strategies, biosensing applications, and key outcomes

Encapsulated CDs	Encapsulation strategy	Biosensing application	Key outcome	References
Blue/red CDs in organosilica nanocapsules (B-CDs@BONs; R-CDs@BONs)	Antibody-modified breakable organosilica nanocapsules; magnetic CD labels	Dual strategies for procalcitonin detection in serum/plasma *via* immunomagnetic separation and homogeneous FRET immunoassay	LOD 0.3 pg mL^−1^ to 0.41 ng mL^−1^; wide ranges; high recoveries	169
Red-emissive CDs in dendritic fibrous nano-silica (CD@DFNS@SH)	Alkyl thiol-functionalized DFNS encapsulation	Turn-on fluorescence detection and remediation of Hg^2+^; biosensing in *Artemia*	LOD 5 nM; adsorption 695 mg g^−1^; recyclable, excitation-independent	170
Grapefruit peel CQDs in silica nanospheres (CQD-SiNP)	Silica nanosphere encapsulation; sandwich immunoassay	Photoluminescence immunoassay for p53 in serum	LOD 2.7 pg mL^−1^; reduced interference; ELISA agreement	162
CDs and Au nanoclusters co-encapsulated in ZIF-8 (ACZ)	ZIF-8 co-encapsulation, acid-responsive dual-emissive ratio sensor	Urease activity monitoring in saliva; a hydrogel-based portable kit	LOD 0.28 U L^−1^; range 1–60 U L^−1^; smartphone integration	163
PEI@CQDs@Ni-MOF composite	MOF-based electrode coating with CQDs and PEI	Label-free electrochemical detection of HER2+ circulating tumor cells	LOD < 1 cell per mL; linear 100–500 cells per mL; high specificity	171
Boronic acid CDs in UiO-66-NH_2_ with zwitterionic polymer (UiO-66-NH_2_/BN-CDs@PSBMA)	MOF encapsulation plus PSBMA antibiofouling grafting	Fluorescence detection of baicalin in complex biological samples	Anti-protein >96.3%; LOD 0.0064 µmol L^−1^; high adsorption	22
N-CDs in amino-modified mesoporous silica with BSA-AuNCs (N-CDs@mSiO_2_-NH_2_@BSA-AuNCs)	Mesoporous silica encapsulation; AuNC coupling	Ratiometric fluorescence detection of CO^2+^ in soils; ML-assisted prediction	Dual emission; LOD 0.74 µM; 10 s detection; *R*^2^ ≈ 0.991	172
Near-IR CDs in polymer dots (Pdots-encapsulated NIR CDs)	ER-targeted polymer dots encapsulation	*In vivo* Cu^2+^ monitoring in cells, zebrafish, and mice	LOD 13 nM; fast 120 s response; selective; biocompatible	164
CDs covalently hybridized with DMSNs (CDs@DMSNs)	*In situ* covalent hybridization forming RTP nanobeads	Time-resolved phosphorescent biosensing and bioimaging; FA receptor targeting	Ultra-long lifetime (1.195 s); time-gated imaging; cell discrimination	136
Nucleoside CDs within DNA hydrogel (ACDs in i-motif hydrogel)	Dual-trigger pH/miRNA-responsive hydrogel releasing ACDs	Electrochemical miRNA sensing; exosome subtype discrimination	Stable probes up to 120 h; 5 min response; 83.33% classification	165
CDs from disposable masks in PVA films (PVA@CDs)	Polymer film integration (encapsulation)	Pathogen sensing (*E. coli*, *S. aureus*) and UV-blocking films	LOD 8–9 CFU per mL; ∼100% UV blocking; stable fluorescence	173
CQDs encapsulated in super-small Pt nanocrystals/graphene (CQD@PDA@PtNCs-NGR)	Core–shell PtNCs templated by CQDs; graphene hybrid	Electrochemical detection of 8-OH-dG in urine; DNA damage linkage	LOD 0.45–0.85 nM; wide linear range; mechanism validation	166
CDs-loaded liposomes with aptamers (CDs-Lip-Apt)	Liposomal encapsulation and release; split-type PEC	Polarity-reversal PEC assay for exosomal MUC1 and PD-L1 proteins	Enhanced accuracy and sensitivity; profiling multiple exosomes	168
CDs/ZIF-8 with PEDOT : PSS and AuNPs (CDs/PEDOT : PSS/ZIF-8)	ZIF-8 carrier; polymer conductivity enhancement; AuNPs	Ultrasensitive ECL detection of miRNA-21 in serum	∼8–10× ECL enhancement; LOD 50 aM; stable and selective	174
CDs@ZIF-8 nanoprobe	ZIF-8 encapsulation with competitive coordination release	Direct, non-enzymatic butyrylcholinesterase assay in clinical sera	LOD 1.5 U L^−1^; accurate in 40 patient samples; mechanism clarified	175
AuNCs/CDs@SiO_2_ core–satellite	Silica-coated CDs with AuNCs; ratio fluorescence	On-site monitoring of chloroquine in creams, serum, and urine	LOD 89.2 nM; portable hydrogel kit; smartphone readout	167
CQDs in DCC-assisted cellulose (CQD/CMC/DCC)	Chemical encapsulation in modified carboxymethyl cellulose	Non-enzymatic *ex vivo* glucose sensing in human serum	LOD 7 nM; dual linear ranges; clinical recovery 100 ± 5%	23

Polymer-based encapsulation strategies broaden the functional scope of CD biosensors by improving biocompatibility and enabling subcellular targeting. Huang *et al.*^164^ encapsulated near-infrared CDs within polymer dots engineered for endoplasmic reticulum localization, enabling rapid and selective monitoring of Cu^2+^ levels in living cells and animal models with minimal cytotoxicity. DNA hydrogels provide another effective encapsulation matrix: Zhang *et al.*^165^ embedded nucleoside-CD probes within hydrogel networks to prevent probe leakage and achieve stable, rapid electrochemical detection of miRNAs and exosome subtypes. Encapsulation techniques also address challenges associated with nonspecific adsorption and sample fouling. A MOF-CD composite coupled with zwitterionic polymer brushes, as reported by Tang *et al.*,^22^ demonstrated strong antibiofouling properties that facilitated selective fluorescence detection of baicalin even in complex herbal extracts and biological fluids.

Encapsulation within hybrid nanocomposites provides yet another pathway for high-performance sensing. Zhang *et al.*^166^ developed CQDs embedded within platinum nanocrystals supported on graphene, a configuration that markedly enhanced electrocatalytic activity and enabled ultrasensitive detection of oxidative DNA damage markers in urine. Encapsulation strategies have also been adapted to practical point-of-care formats. Wang *et al.*^167^ designed silica-coated CDs arranged in core-satellite structures with gold nanoclusters to produce ratiometric fluorescence sensors for chloroquine detection. These sensors were combined with smartphone-enabled hydrogel test kits for accessible, on-site monitoring. Similarly, Patra *et al.*^23^ encapsulated CQDs in cellulose matrices to achieve robust, non-enzymatic glucose sensing, demonstrating high operational stability and successful validation with clinical samples.

**Fig. 16:**
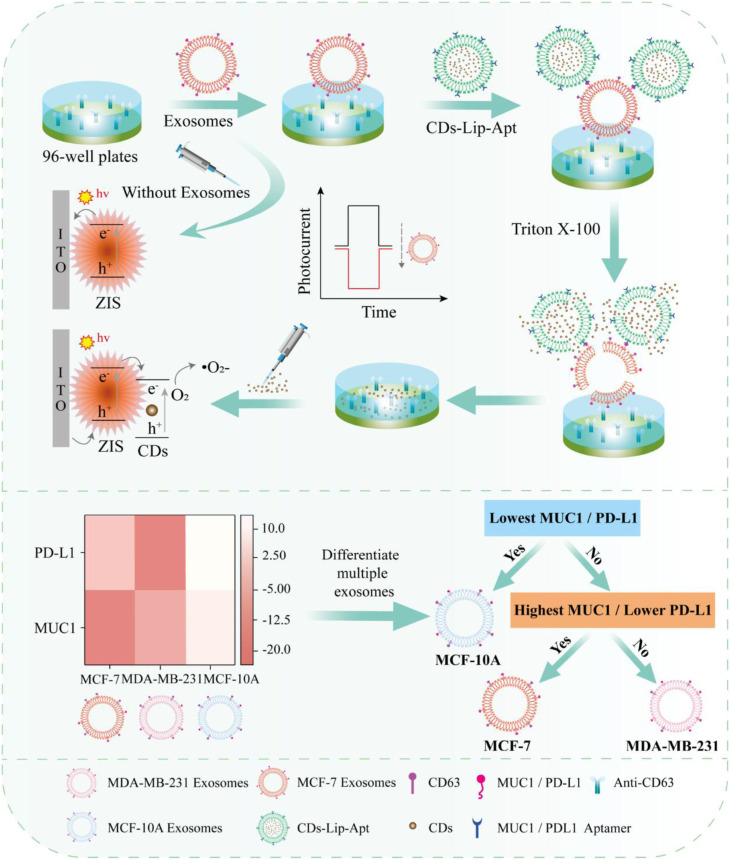
Polarity-reversal PEC sensing platform for exosome assays based on CDs-induced ZIS photocurrent polarity-reversal, reproduced from ref. 168 with permission from Elsevier,^168^ Copyright 2026.

A particularly innovative encapsulation-based biosensing platform was developed by Hou *et al.*,^168^ who introduced a polarity-reversal photoelectrochemical (PEC) system for exosome analysis, illustrated in Fig. 16. In this design, CDs were encapsulated within liposomes modified with aptamers (CDs-Lip-Apt). Upon binding and subsequent lysis of target exosomes, the released CDs served as photoactive elements that triggered photocurrent polarity reversal in a ZnIn_2_S_4_ (ZIS)-modified electrode. This polarity-switching mechanism significantly enhanced anti-interference performance, improved detection sensitivity, and enabled precise quantification of exosomal biomarkers such as MUC1 and PD-L1. The system underscores how encapsulation not only stabilizes CDs but also enables advanced signal-transduction strategies for clinical diagnostic applications.

Overall, these advancements demonstrate that encapsulation is far more than a protective measure; it is a fundamental design principle that elevates the stability, sensitivity, and selectivity of CD-based biosensors. By mitigating environmental interference, enabling targeted interactions, and supporting diverse transduction mechanisms, encapsulated CDs continue to expand the scope and reliability of biosensing technologies across biomedical and analytical settings.

### 4.5 Other applications

Beyond their widely explored applications in drug delivery, bioimaging, theranostics, and biosensing, encapsulated CDs have demonstrated notable versatility across a broader range of biomedical technologies. Recent studies reveal that integrating CDs into hydrogels, protein matrices, and inorganic frameworks can unlock enhanced therapeutic behaviors, including controlled release, enzymatic mimicry, and synergistic biological activity. For example, copper-doped CDs embedded within biocompatible hydrogels exhibit intrinsic peroxidase-like activity, enabling stimuli-responsive biofilm disruption and promoting accelerated wound healing.^176^ Similarly, injectable chitosan-based hydrogels containing CDs with dual enzyme-mimicking properties have demonstrated potent antibacterial efficacy in infected wound sites, underscoring how polymeric encapsulation can extend retention time and amplify therapeutic impact.^177^ Plant-derived CDs have also been encapsulated within sodium alginate hydrogels alongside baicalein, producing sustained antioxidant and antibacterial activity that significantly improved wound closure *in vivo*.^178^ Protein-based systems, such as egg-white hydrogels crosslinked with CDs, have further demonstrated enhanced mechanical strength and biocompatibility, supporting applications in tissue engineering.^179^

Inorganic encapsulation approaches also broaden the functional landscape of CD-based therapeutics. Encapsulation within zeolitic imidazolate framework-8 (ZIF-8), for instance, yields nanoplatforms capable of providing multi-antioxidant protection, effectively safeguarding renal epithelial cells from oxidative stress and demonstrating the cytoprotective potential of MOF-derived systems.^180^ Hybrid constructs that combine CDs with magnetic nanoparticles or upconversion nanomaterials have additionally been developed for photodynamic therapy, enabling deeper tissue treatment through near-infrared light activation.^181^ These examples highlight how encapsulation not only stabilizes CDs but also confers advanced functionalities, such as pH responsiveness, enzyme mimicry, or multimodal therapeutic action, thereby broadening their impact across diverse biomedical domains.

**Fig. 17:**
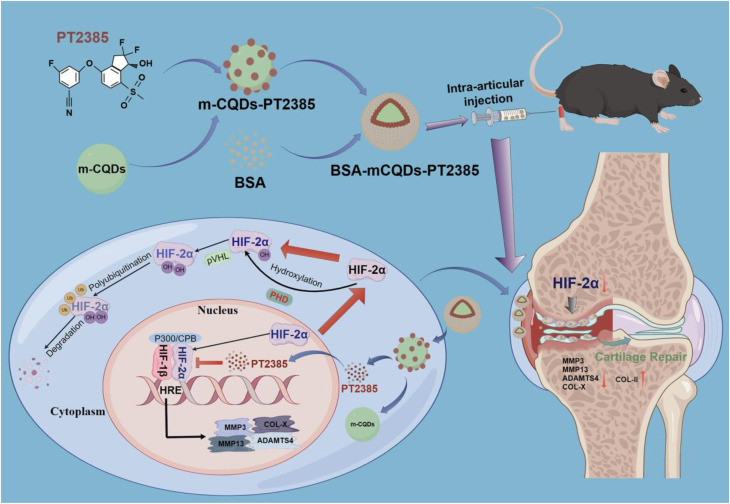
Schematic of BCP encapsulation: PT2385-loaded m-CQDs coated with BSA enable pH-responsive release, cartilage penetration, and HIF-2α inhibition to protect against early osteoarthritis, reproduced from ref. 182 with permission from Elsevier,^182^ Copyright 2024.

An example of these multifunctional systems is the bovine serum albumin-carbon quantum dot-PT2385 (BCP) nanoplatform designed by Zhang *et al.* for osteoarthritis therapy.^182^ As illustrated in Fig. 17, PT2385, a small-molecule inhibitor of hypoxia-inducible factor-2α (HIF-2α), was complexed with *m*-phenylenediamine-derived carbon quantum dots and subsequently encapsulated within a BSA shell. This architecture enabled efficient penetration into cartilage tissue and exhibited pH-responsive release behavior, ensuring that drug liberation coincided with the acidification characteristic of osteoarthritic progression. Upon entry into chondrocytes, PT2385 disrupted the HIF-2α/HIF-1β interaction, promoting HIF-2α degradation and thereby reducing the expression of downstream catabolic mediators such as matrix metalloproteinases (MMPs) and ADAMTS proteases. This targeted inhibition effectively protected against early cartilage degeneration, demonstrating how encapsulation transforms a systemically limited inhibitor into a localized, disease-responsive therapeutic. The BCP system thus exemplifies the broader principle that encapsulation can endow CDs with the capacity to deliver small molecules in a controlled, tissue-specific, and environmentally responsive manner, opening new avenues for treating complex degenerative diseases.

Overall, encapsulation approaches not only enhance the biochemical stability and functional performance of CDs but also enable their integration into sophisticated therapeutic architectures. These emerging systems represent a growing class of adaptable nanocarriers with significant potential for next-generation biomedical applications, ranging from regenerative medicine to inflammation control and targeted disease modulation.

**Fig. 18:**
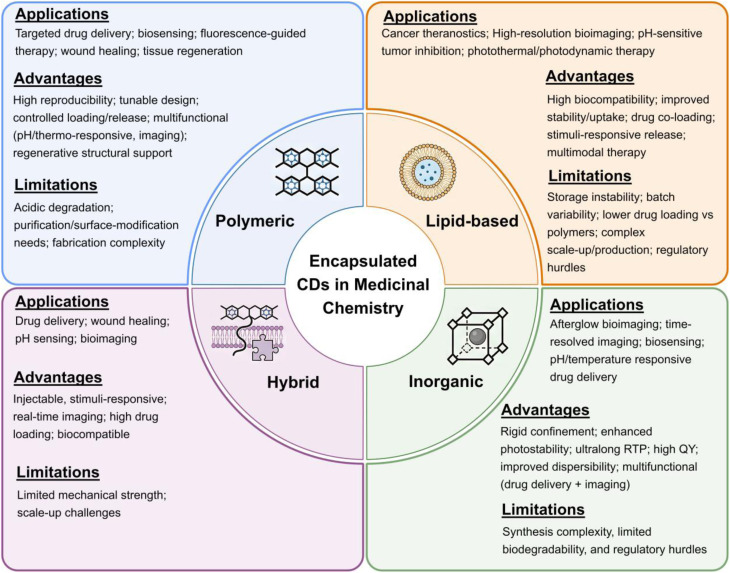
Schematic summary of encapsulated carbon dot systems. The figure categorizes major encapsulation strategies (polymeric, lipid-based, inorganic, and hybrid) and maps them to their dominant biomedical applications and key advantages and limitations.

To facilitate rapid comparison of the information presented in Tables 1–5, the major encapsulation strategies of CDs and their applications, advantages, and limitations have been summarized in Fig. 18.

## 5 Challenges and future perspectives

**Fig. 19:**
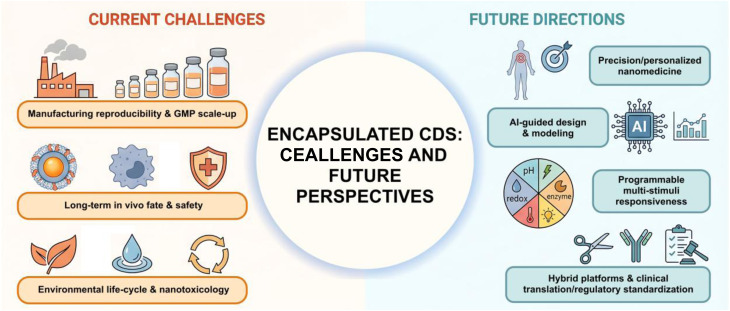
Main challenges and future directions in encapsulation of CDs for medicinal applications. Challenges (left, orange): manufacturing reproducibility & GMP scale-up, long-term *in vivo* fate & safety, and environmental life-cycle & nanotoxicology. Future directions (right, blue): precision/personalized nanomedicine, AI-guided design & modeling, programmable multi-stimuli responsiveness, and hybrid platforms & clinical translation/regulatory standardization.

Encapsulated CDs have demonstrated considerable promise across imaging, drug delivery, and theranostic applications; however, translation beyond laboratory-scale demonstrations requires a shift from performance-driven optimization toward mechanistic clarity and regulatory-aligned development. Rather than reiterating general calls for “further improvement,” this section outlines concrete research priorities organized around three domains: unresolved mechanistic questions, priority material platforms, and translational bottlenecks. Fig. 19 summarizes the principal challenges (orange panel, left) and future directions (blue panel, right) discussed below.

### 5.1 Unresolved mechanistic questions

Unresolved mechanistic questions remain a central barrier to predictive encapsulation design. A central priority for the field is establishing quantitative structure–property relationships that explain how encapsulation alters CD photophysics and biological behavior. Although confinement within polymeric, lipid, or inorganic matrices frequently enhances stability and afterglow performance, the evolution of surface states under encapsulation remains insufficiently understood. Systematic spectroscopic investigations are needed to determine how matrix rigidity, interfacial bonding, and microenvironment polarity influence radiative *versus* nonradiative decay pathways. Without such mechanistic resolution, rational prediction of emission retention after encapsulation remains limited.

The long-term stability of heteroatom doping under physiological conditions represents a second critical knowledge gap. Nitrogen-, sulfur-, and phosphorus-doped CDs often underpin enhanced fluorescence or catalytic performance, yet the structural integrity of dopant sites during prolonged exposure to biological fluids, enzymatic activity, and oxidative stress is rarely evaluated. Longitudinal degradation studies incorporating real-time spectroscopic monitoring and mass spectrometric analysis are required to determine whether dopant leaching, oxidation, or surface reconstruction occurs *in vivo*.

Furthermore, encapsulation-induced modifications to protein corona formation, cellular uptake pathways, and intracellular trafficking remain poorly quantified. Comparative studies across material classes using standardized biological models are essential to disentangle matrix-driven effects from intrinsic CD behavior. Establishing predictive correlations between encapsulation architecture and pharmacokinetic profiles will be pivotal for advancing design beyond empirical optimization.

### 5.2 Priority material platforms

Priority material platforms must balance optical stability with controlled biodegradability and scalable manufacturing. Future research should prioritize material platforms that balance optical stability with controlled biodegradability and clearance. Hybrid polymer–inorganic architectures represent a promising direction, as they combine structural confinement with tunable degradation pathways. However, systematic investigation of their breakdown products, clearance kinetics, and long-term biocompatibility remains necessary before clinical consideration.

Biodegradable inorganic hosts constitute another strategic opportunity. While silica and zeolite frameworks provide excellent photostability, their limited degradability constrains *in vivo* applicability. The development of transient or bioresorbable inorganic matrices with predictable dissolution behavior could reconcile optical robustness with physiological safety.

Membrane-inspired multifunctional systems also warrant focused investigation, particularly regarding long-term immune interactions and reproducibility of membrane sourcing. Standardized protocols for membrane isolation, characterization, and storage are required to reduce variability and facilitate comparative evaluation across laboratories.

Across all material classes, emphasis should shift from increasing multifunctionality toward optimizing stability-degradability balance and manufacturing simplicity. Demonstrating predictable degradation kinetics and clearance profiles will be as critical as achieving high quantum yield or targeting efficiency.

### 5.3 Translational and manufacturing bottlenecks

Translational and manufacturing bottlenecks represent the final critical barrier between laboratory validation and clinical implementation. Scalable and reproducible manufacturing remains one of the most significant barriers to clinical translation. Many encapsulation strategies rely on multistep synthesis routes, solvent-sensitive assembly processes, or post-synthetic modifications that are difficult to standardize under good manufacturing practice (GMP) conditions. Establishing reproducibility benchmarks, including batch-to-batch variability in size distribution, encapsulation efficiency, surface chemistry, and emission properties, should become a field-wide priority.

Long-term degradation profiling and pharmacokinetic evaluation are equally urgent. Comprehensive *in vivo* studies must quantify biodistribution, metabolic pathways, clearance mechanisms, and potential chronic accumulation. Particular attention should be given to hybrid systems, where degradation products from multiple components may interact in unpredictable ways.

The absence of standardized *in vivo* evaluation protocols further complicates regulatory assessment and cross-study comparability. To strengthen methodological transparency and translational benchmarking, we propose a minimum reporting framework for encapsulated carbon dot systems intended for *in vivo* application. This framework defines essential baseline characterization elements that should accompany preclinical investigations.

First, core physicochemical properties must be comprehensively documented, including particle size distribution (mean diameter and polydispersity index), morphology (electron microscopy), surface charge (zeta potential), quantum yield, and structural confirmation of encapsulation. Measurement conditions and batch-to-batch variability should be explicitly reported to ensure reproducibility.

Second, encapsulation performance under physiologically relevant conditions should be evaluated. This includes encapsulation efficiency, loading capacity where applicable, and release kinetics in buffered saline and serum-containing media at 37 °C. For stimulus-responsive systems, trigger-specific release profiles must be quantitatively compared with physiological controls to demonstrate functional selectivity.

Third, pharmacokinetic and clearance profiling should be incorporated into *in vivo* studies. Circulation half-life, organ biodistribution over defined time intervals, routes of elimination, and long-term retention must be quantified using appropriate imaging or analytical methods. Without such data, claims of biocompatibility or translational readiness remain incomplete.

Fourth, toxicity assessment should extend beyond short-term cytotoxicity assays. Recommended evaluation includes hematological parameters, liver and kidney function markers, histopathological analysis, complement activation testing, and inflammatory cytokine profiling. Where hybrid or inorganic systems are employed, degradation products and potential metal ion release must also be considered.

Finally, statistical reproducibility metrics should be transparently reported, including the number of biological replicates, independent synthesis batches, and variance for key physicochemical and biological measurements. Demonstrating reproducibility across production batches is particularly critical for multifunctional or multicomponent architectures.

Adoption of such a structured reporting baseline would facilitate meaningful comparison across material platforms, support meta-analytical evaluation, and improve alignment with regulatory expectations. Early engagement with agencies such as the FDA and EMA will remain essential to define approval pathways tailored to multifunctional nanotherapeutics; however, consistent reporting standards represent a prerequisite for constructive regulatory dialogue.

Advancing encapsulated CD systems toward clinical relevance demands coordinated progress in mechanistic understanding, material innovation, and translational infrastructure. Improvements in optical performance alone are insufficient; long-term stability, degradability, reproducibility, and regulatory feasibility must be addressed concurrently. By aligning future research with these priorities, the field can transition from exploratory demonstrations to robust, clinically viable nanomedicine platforms.

From our assessment, the field is approaching a point of performance saturation in certain areas. Incremental increases in multifunctionality, through increasingly complex hybrid architectures, do not necessarily translate into improved translational feasibility. While rigid inorganic confinement strategies provide exceptional photophysical stability, their limited biodegradability may impose fundamental constraints on long-term clinical adoption unless degradable alternatives are developed. Similarly, membrane-cloaked and highly engineered hybrid systems offer impressive targeting capabilities but introduce reproducibility and sourcing challenges that may hinder regulatory approval.

In our view, the most promising direction lies in rationally simplified hybrid platforms that balance optical robustness with predictable degradation and scalable manufacturing. Rather than maximizing functional integration, future efforts should prioritize mechanistic clarity, degradability control, and reproducible synthesis. Platforms that achieve moderate multifunctionality with high reliability are more likely to reach clinical relevance than highly complex systems optimized primarily for proof-of-concept performance.

## 6 Conclusion

In this review, we have highlighted how encapsulation fundamentally enhances the medicinal potential of carbon dots by improving their stability, biodistribution, optical durability, and therapeutic performance. Strategies employing polymeric, lipid-based, inorganic, and hybrid matrices not only protect and stabilize CDs but also enable targeted delivery, controlled release, and the integration of imaging and therapeutic functions, advancing their roles in drug delivery, bioimaging, theranostics, and biosensing. Moving forward, overcoming challenges in reproducible synthesis, long-term biosafety, and scalable production, alongside establishing standardized characterization protocols, will be crucial for clinical translation. As advances in materials science, computational design, and biomedical engineering converge, encapsulated CDs are well-positioned to underpin the next generation of intelligent, personalized nanomedicine.

## Conflicts of interest

There are no conflicts to declare.

## Data Availability

No data was used for the research described in the article.
